# Human mRNA in saliva can correctly identify individuals harboring acute infection

**DOI:** 10.1128/mbio.01712-23

**Published:** 2023-11-09

**Authors:** Qing Yang, Nicholas R. Meyerson, Camille L. Paige, James H. Morrison, Stephen K. Clark, Will T. Fattor, Carolyn J. Decker, Halley R. Steiner, Elena Lian, Daniel B. Larremore, Rushika Perera, Eric M. Poeschla, Roy Parker, Robin D. Dowell, Sara L. Sawyer

**Affiliations:** 1 BioFrontiers Institute, University of Colorado Boulder, Boulder, Colorado, USA; 2 Department of Molecular, Cellular, and Developmental Biology, University of Colorado Boulder, Boulder, Colorado, USA; 3 Darwin Biosciences, Inc., Boulder, Colorado, USA; 4 Division of Infectious Diseases, Department of Medicine, University of Colorado School of Medicine, Aurora, Colorado, USA; 5 Department of Biochemistry, University of Colorado Boulder, Boulder, Colorado, USA; 6 Howard Hughes Medical Institute, Chevy Chase, Maryland, USA; 7 Center for Vector-Borne Infectious Diseases and Department of Microbiology, Immunology and Pathology, Colorado State University, Fort Collins, Colorado, USA; 8 Department of Computer Science, University of Colorado Boulder, Boulder, Colorado, USA; 9 Santa Fe Institute, Santa Fe, New Mexico, USA; Washington University in St. Louis School of Medicine, St. Louis, Missouri, USA

**Keywords:** innate immunity, biomarkers, infectious disease screening, saliva, host gene transcripts, transcriptional response to infection

## Abstract

The COVID-19 pandemic demonstrated the poor ability of body temperature to reliably identify SARS-CoV-2-infected individuals, an observation that has been made before in the context of other infectious diseases. While acute infection does not always cause fever, it does reliably drive host transcriptional responses as the body responds at the site of infection. These transcriptional changes can occur both in cells that are directly harboring replicating pathogens and in cells elsewhere that receive a molecular signal that infection is occurring. Here, we identify a core set of approximately 70 human genes that are together upregulated in cultured human cells infected by a broad array of viral, bacterial, and fungal pathogens. We have named these “core response” genes. In theory, transcripts from these genes could serve as biomarkers of infection in the human body, in a way that is agnostic to the specific pathogen causing infection. As such, we perform human studies to show that these infection-induced human transcripts can be measured in the saliva of people harboring different types of infections. The number of these transcripts in saliva can correctly classify infection status (whether a person harbors an infection) 91% of the time. Furthermore, in the case of SARS-CoV-2 specifically, the number of core response transcripts in saliva correctly identifies infectious individuals even when enrollees, themselves, are asymptomatic and do not know they are infected.

**IMPORTANCE:**

There are a variety of clinical and laboratory criteria available to clinicians in controlled healthcare settings to help them identify whether an infectious disease is present. However, in situations such as a new epidemic caused by an unknown infectious agent, in health screening contexts performed within communities and outside of healthcare facilities or in battlefield or potential biowarfare situations, this gets more difficult. Pathogen-agnostic methods for rapid screening and triage of large numbers of people for infection status are needed, in particular methods that might work on an easily accessible biospecimen like saliva. Here, we identify a small, core set of approximately 70 human genes whose transcripts serve as saliva-based biomarkers of infection in the human body, in a way that is agnostic to the specific pathogen causing infection.

## INTRODUCTION

There are a variety of clinical and laboratory criteria available to clinicians in controlled healthcare settings to help them identify whether an infectious disease is present. However, in situations such as a new epidemic caused by an unknown infectious agent, in health screening contexts performed within communities and outside of healthcare facilities or in battlefield or potential biowarfare situations, this gets more difficult. Pathogen-agnostic methods for rapid screening and triage of large numbers of people for infection status are needed, in particular methods that might work on an easily accessible biospecimen like saliva.

Infection drives the transcription of host genes required to execute various immune responses. These transcriptional changes can occur both in cells that are harboring replicating pathogens and in cells elsewhere that receive a molecular signal that infection is occurring. During infection, some of these upregulated transcripts and proteins (collectively, “biomarkers” of infection) will make their way into the bloodstream, either as waste products from destroyed cells or because blood-circulating cells, themselves, are reacting to signals of infection. Indeed, numerous previous studies have monitored human proteins and transcripts in blood that change with infection, infectious disease severity, and symptom status (1–19). The utility of host-derived infection biomarkers would be expanded if two notional criteria could be met: (i) biomarkers could be identified that are part of a core host response to all or most infections, so that infection could be detected even when caused by new, unknown, or unsuspected pathogens, and (ii) biomarkers of infection could be measured in a non-invasive biological sample (e.g. saliva).

Many studies have catalogued human genes that are upregulated in response to infection with different specific pathogens. Our first goal was to identify a set of human genes that are commonly upregulated by diverse pathogens. While genes in certain innate immunity pathways, such as the interferon response, theoretically should respond to many types of infection, the interferon response is bifurcated with numerous different interferons and signaling pathways (20). Thus, we decided to not make assumptions based on knowledge of known innate immune pathways and instead to derive this transcript list *de novo* using an assemblage of published data sets. We identified a core set of 69 human genes that are commonly upregulated in cultured human cells infected with an array of different pathogens (fungal, viral, and bacterial). Then, we use multiple human cohorts to show that the transcripts from these genes can be measured in human saliva and can be used to classify human infection status (i.e., infected or not infected) with high specificity and sensitivity. Together, these findings demonstrate the presence of molecules in saliva that reflect the activation of the human innate immune system.

## RESULTS

### Identification of human genes upregulated by diverse pathogens

**Fig 1:**
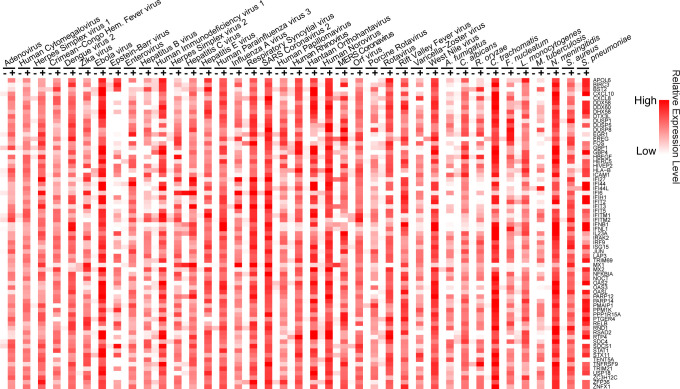
Identification of 69 human genes upregulated in cells infected with a broad range of pathogens. Heatmap summarizing abundance of mRNA transcripts from RNA-seq data. Each row represents 1 of the 69 core response genes. Each column represents the average expression across all mock (−) or infected (+) replicates combined from all studies on a given pathogen [Z-score of log10 (TPM + 1); TPM, transcripts per million]. The studies used are summarized in Table S1. The core response genes are ordered alphabetically.

We identified and downloaded 71 published data sets that measured the transcriptional response of human cells to infection. These data sets each involved human cells, grown in culture, which were infected with a variety of pathogens (29 viruses, 7 bacteria, and 3 fungi). Some pathogens were represented in more than one data set (Table S1). Each of the 71 studies generated matched RNA sequencing (RNA-seq) data for infected and mock-infected human cells, usually in multiple replicates (*n* = 387 replicates in all). Raw RNA-seq data were retrieved from the NCBI short-read archive and mapped to the reference human genome (GRCh38). For each data set, the infected replicates were size normalized and compared to the matching mock replicates via differential expression analysis. The differentially expressed genes identified in each study were then compared across studies, so that we could identify genes significantly upregulated in ≥80% of the 71 data sets. Transcripts from 69 genes met this criterion (shown visually in Fig. 1, gene list in Table S2). As expected, many of these 69 genes belong to pathways related to cellular innate immune functions and interferon responses (gene ontology analysis shown in Fig. S1). We originally designed the 80% threshold to account for errors in the data sets, the many variables between the data sets, and stochastic effects. Nevertheless, of the 69 genes identified, transcripts from 7 of the genes (DDX58, FOS, IFIT2, IRAK2, IRF9, PMAIP1, and LAP3) were significantly upregulated in 95% of the data sets. Because of the approach that we took, we cannot say for sure that all 69 human genes are transcriptionally activated by every pathogen, but rather that most of these genes are induced by most pathogens (Fig. 1). Ultimately, we were seeking to identify the “core” or most fundamental cell-based transcriptional response to infection stress. Next, we put the gene set we have identified through four critical tests of this premise.

**Fig 2:**
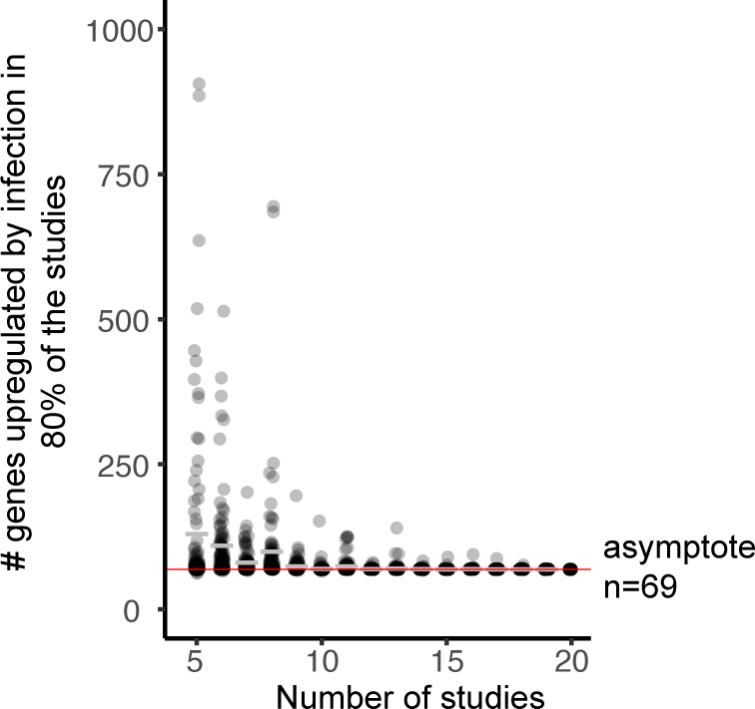
The identified transcript set represents a core response to infection. From each of the 71 *in vitro* infection data sets, we curated a list of significantly upregulated genes. We then compared these genes between groups of data sets, with 100 random combinations compared for each number of data sets (X axis). The Y axis shows the number of commonly upregulated genes identified (upregulated in 80% or more of the data sets). Gray bars are the mean values, and a line is placed at Y axis = 69. The number of commonly upregulated genes stabilizes at *n* = 69 genes.

First, we address the fact that the genes identified were only required to be upregulated in 80% of the data sets analyzed. If these transcripts represent a core transcriptional response to infection, we should identify the same gene set consistently regardless of the specific data sets used to find them. On the other hand, if these 69 genes do not represent a core transcriptional response to infection, then we should find different genes reaching the >80% threshold as we alter the composition of the data sets. To test this premise, we used a bootstrapping approach, selecting randomly 5 of the 71 data sets, multiple times, and in each iteration identifying genes significantly upregulated by infection across 80% of the data sets. This bootstrapping was then repeated with increasing numbers of data sets included (5, 6, 7, etc.), and in each case, the shared set of upregulated genes was recomputed. Using this approach, we found that the number of upregulated genes stabilized after ~17 studies was included, regardless of which 17 studies were selected (Fig. 2). The set of commonly upregulated genes reached an asymptotic value of 69 genes; the addition of more data sets did not add or subtract genes from the set. This suggests that we have identified a core and connected transcriptional response to infection. Extrapolation of the curve indicates that the set of core response genes will remain basically constant, even if many more data sets are added.

**Fig 3:**
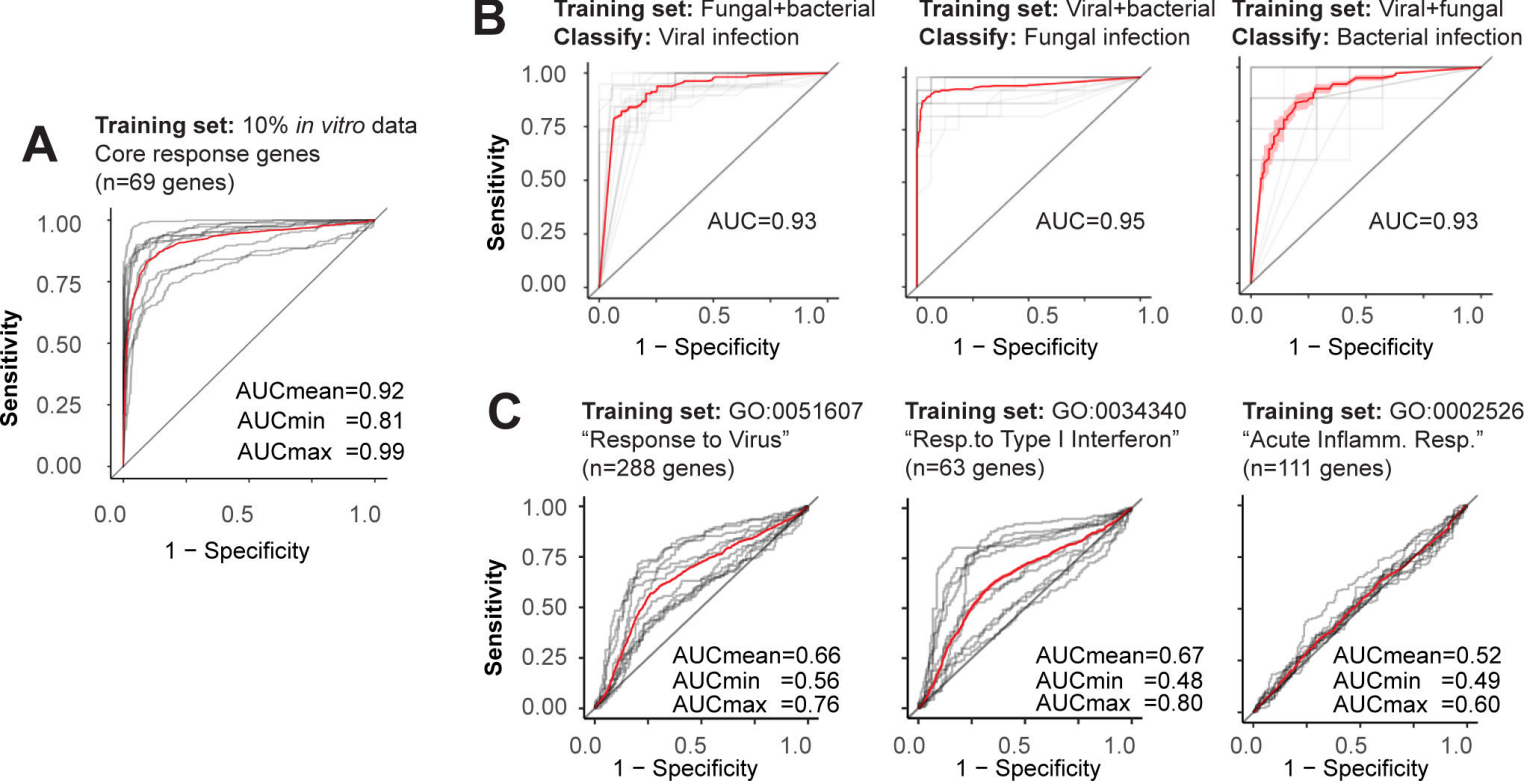
Performance of various infection classifiers. ROC curves of various logistic regression models were established using the expression levels of the 69 core response genes. The AUC is summarized in each graph. Each cross-validation is repeated 10 times, with gray lines indicating each replicate of cross-validation; red curve summarizes the average ROC curve. (**A**) A logistic regression model was trained on data from 10% of the 387 samples from the 71 *in vitro* data sets, then used to classify the other 90% of the samples as mock infected or infected. (**B**) Cross-validation analyses between different types of infections. In each case, the logistic regression model was trained on infections of two types (top of graph) and used to classify human cells as having been infected or not with the third type of pathogen, based solely on the expression level of the core response genes. (**C**) The identical analysis as panel A, except models were trained on genes from indicated gene ontology terms, instead of on the 69 core response genes identified here.

As a second test, we built a classifier from the identified core gene set (*n* = 69 genes), with the goal of classifying human cells as having been infected or not based solely on the number of these transcripts present. Using the 387 independent experimental replicates from the 71 *in vitro* infection data sets, we utilized cross-validation to explore the effectiveness of a logistic regression model. Specifically, we established the logistic regression classifier using expression data from the 69 core response genes from a randomly selected 10% of the 387 replicates (much less than what is typically used in 10-fold cross-validation experiments, done to emphasize the discriminatory power). Using this, we classified the remaining 90% of the 387 replicates as infected or not. This cross-validation was repeated 10 times, and the accuracy of classification is summarized via receiver operating characteristic (ROC) curve (Fig. 3A, with precision-recall curves shown in Fig. S2A). Overall, the cross-validation resulted in a mean area under the curve (AUC) of 0.92. The worst outcome of the 10 repeats had an AUC of 0.81, and the best, an AUC of 0.99. We conclude that tissue culture samples can be classified as having been infected or not, with high sensitivity and specificity, based solely on the abundance in the sample of transcripts from these 69 human genes. This is true, even though these 71 data sets represent a diverse array of human pathogens.

As a third test, we sought to confirm whether the identified transcriptional response is reliably induced by diverse pathogen types. If so, we reasoned that models trained on the abundance of these core response transcripts in samples infected with one pathogen type (fungal, viral, or bacterial) should correctly categorize samples from other pathogen categories as infected or not. Thus, we trained a logistic regression classifier using core response gene expression data from two of the three pathogen types (fungal, viral, or bacterial) and then classified all experimental replicates of the remaining type as infected or not. Here, realizing that viruses are over-represented within the 71 data sets, we randomly down sampled the viral infection data to match the sample size of the other data sets, before using the subset for training. The cross-validation worked equally well when virus-infected data sets were not down sampled. This cross-pathogen validation yielded ROC curves with mean AUCs ranging from 0.93 to 0.95 (Fig. 3B, with precision-recall curves shown in Fig. S2B). Thus, even when the training data are derived solely from cells infected with two classes of pathogen (fungal, viral, or bacterial), a logistic regression classifier can correctly categorize cells as having been infected with a third class of pathogen. We conclude that these 69 genes represent a meaningful and informative response to infection that extends across pathogen types.

Finally, we tested whether we had simply rediscovered a set of immune-related genes already known to correspond to host responses to infection, interferon, or inflammation. We next tested our set of core response genes versus alternative, similar sets of known genes. We again performed the analysis as shown in Fig. 3A, except we trained the classifier on the expression levels of genes in relevant gene ontology (GO) terms (shown at the top of graphs; Fig. 3C) instead of the core response genes. None of the examined gene sets were able to distinguish infected and non-infected tissue culture samples to the same degree as the core response genes. We tried other GO terms (not shown), and none performed better than the examples shown. Thus, the 69 human genes we have identified have significantly more power to classify cell lines experiencing pathogen infection than other previously defined human gene sets.

For the remainder of this study, we investigate transfer learning approaches to see if abundance of transcripts from these 69 core response genes (identified from *in vitro* data) can classify living humans as infected or not.

### Core response transcripts can be measured in blood and saliva of infected individuals

The first task was to determine if these gene transcripts, upregulated by infection in tissue culture, are also upregulated by infection in living humans. Saliva purportedly contains similar molecules as found in blood (21), and so we wondered whether core response transcripts could be measured in a highly accessible biospecimen like saliva. Saliva is not commonly used in the literature or in clinical diagnostics, so we started with assessing feasibility. We obtained saliva samples from 15 healthy individuals (SS01–SS15; Table 1) and 3 individuals hospitalized and recently diagnosed with SARS-CoV-2 viral infection (enrollees SS19–SS21; Table 1). Total RNA was prepared from each of these human saliva samples, followed by depletion of bacterial and human ribosomal RNA. We were able to isolate high-integrity RNA from saliva samples (Fig. S3). Libraries were sequenced with high-throughput short-read sequencing.

**TABLE 1:** Saliva cohort 1, healthy enrollees and enrollees diagnosed with SARS-CoV-2

Enrollee	Collection date	Diagnosis/infectious agent	Study site
SS01–15	March-Dec 2019	15 apparently healthy enrollees	University of Colorado Hospital, Anschutz Medical Campus; University of Colorado Boulder
SS19–21	May 2020	3 patients being treated for SARS-CoV-2 infection. Saliva samples were taken 4–7 days after diagnosis.	University of Colorado Hospital, Anschutz Medical Campus

**Fig 4:**
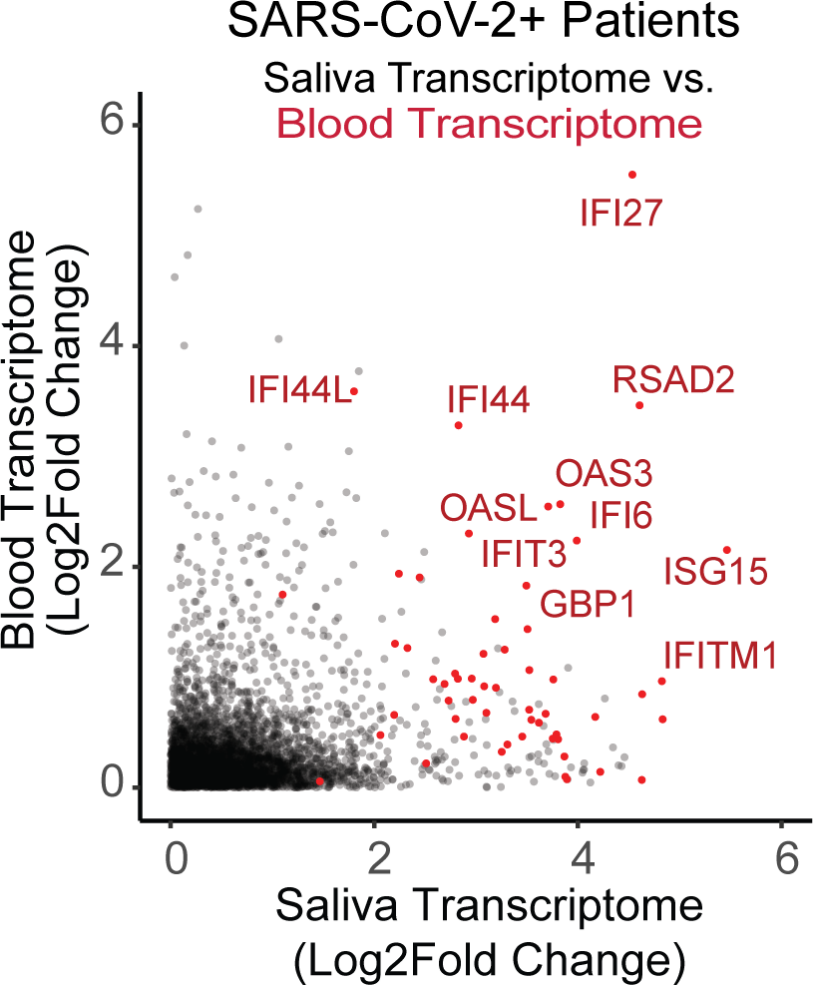
Transcripts from core response genes can be detected in blood and saliva. On the X-axis, the abundance of human transcripts in the saliva of three SARS-CoV-2 patients relative to uninfected control individuals (enrollees shown in Table 1). On the Y-axis is the similar analysis, performed on blood from infected and uninfected individuals in a different SARS-CoV-2 cohort (22). Each dot is a gene, and the core response genes are shown in red. There are 64 red points shown here; the other five genes were downregulated in either blood or saliva. By the criteria of fold change > 2 (or log 2 fold change > 1) in infected individuals versus uninfected, many core response genes are significantly upregulated in blood and/or saliva.

For each human gene, we calculated the enrichment of transcripts in saliva from infected individuals relative to healthy ones (X-axis in Fig. 4). We then color coded the 69 core response genes with red dots. Because of the inherent noise from sequencing, many genes will (by chance) have a fold change value >0 in infected individuals. Therefore, for a gene to be considered significantly upregulated, we require fold change value of >2 (or log 2 fold change of >1). By this criterion, transcripts from 64 out of 69 core response genes are significantly upregulated in saliva of infected individuals. We conclude that core response transcripts are, indeed, measurable in human saliva and significantly upregulated by infection with SARS-CoV-2.

We next compared saliva to blood. We analyzed levels of core response transcripts in the blood of people infected with SARS-CoV-2, using data from the COVIDome database (22). Using the COVIDome data, we calculated the enrichment of transcripts from each human gene in the blood of infected (*n* = 105) versus healthy enrollees (Y-axis in Fig. 4). We found an enrichment of transcripts from 21 out of 69 (30%) of the core response genes in the blood of infected individuals versus healthy ones; this is fewer than the number elevated in saliva (where 64 out of 69 were at significantly higher levels). Based on the limited analysis presented here, it is possible that core response transcripts are even more reliably detected in saliva than in blood, but more research into this comparison will be needed. In particular, note the small sample size for saliva here (*n* = 3) and the fact that the blood and saliva analyzed here are not matched from the same patients. Matched blood and saliva samples from individuals with an array of infections could be used to systematically address whether saliva is truly a better biospecimen than blood for detecting infection-induced host transcripts. Given the encouraging results from this pilot cohort, for the rest of this study, we focus on human saliva.

### The abundance of core response transcripts in saliva can classify human infections

Next, we investigated these infection-induced transcripts in the saliva of individuals with a broader range of infections. In addition to samples included in Table 1, we enrolled another five individuals recently diagnosed with a variety of infections. One had been diagnosed with *Vibrio cholerae* bacterial infection (SS16), one with *Staphylococcus aureus* bacterial infection (SS17), and one with varicella-zoster virus infection (SS18). Two additional enrollees were university affiliates from whose saliva we were able to map reads corresponding to common respiratory pathogens (SS22, CoV-NL63 seasonal coronavirus; SS23, respiratory syncytial virus [RS]). In total, 23 enrollees donated saliva (cohort 2, Table 2), from which total RNA was prepared and subjected to high-throughput short-read sequencing.

**TABLE 2:** Saliva cohort 2, individuals harboring diverse infections

Enrollee	Collection date	Diagnosis/infectious agent with clinical notes where available	Study site
SS01–15	March-Dec 2019	15 apparently healthy enrollees. Also included in cohort 1.	University of Colorado Hospital, Anschutz Medical Campus; University of Colorado Boulder
SS16	September 2019	Patient with gastroenteritis caused by *Vibrio cholerae* bacteria. Received one dose of Cipro and ceftriaxone before saliva sample taken.	University of Colorado Hospital, Anschutz Medical Campus
SS17	September 2019	Patient with methicillin-resistant *Staphylococcus aureus* bacteremia, C5-C7 osteomyelitis/discitis, prevertebral fluid concerning for abscess. No erythema noted in mouth/pharynx. Pulmonary effort is normal and breath sounds normal.	
SS18	September 2019	Patient with varicella-zoster virus meningitis. Herpes zoster. Meningitis, involving left V1-V2 dermatome without ocular involvement. Lungs CTAB, no wheezes, rales or rhonchi, normal effort.	
SS19–21	May 2020	3 patients being treated for SARS-CoV-2 infection. Saliva samples were taken 4–7 days after diagnosis. Also included in cohort 1.	
SS22	January 2020	University affiliate whose saliva contained RNAseq reads mapping to seasonal coronavirus CoV-NL63.	University of Colorado Boulder
SS23	February 2020	University affiliate whose saliva contained RNAseq reads mapping to respiratory syncytial virus.	

**Fig 5:**
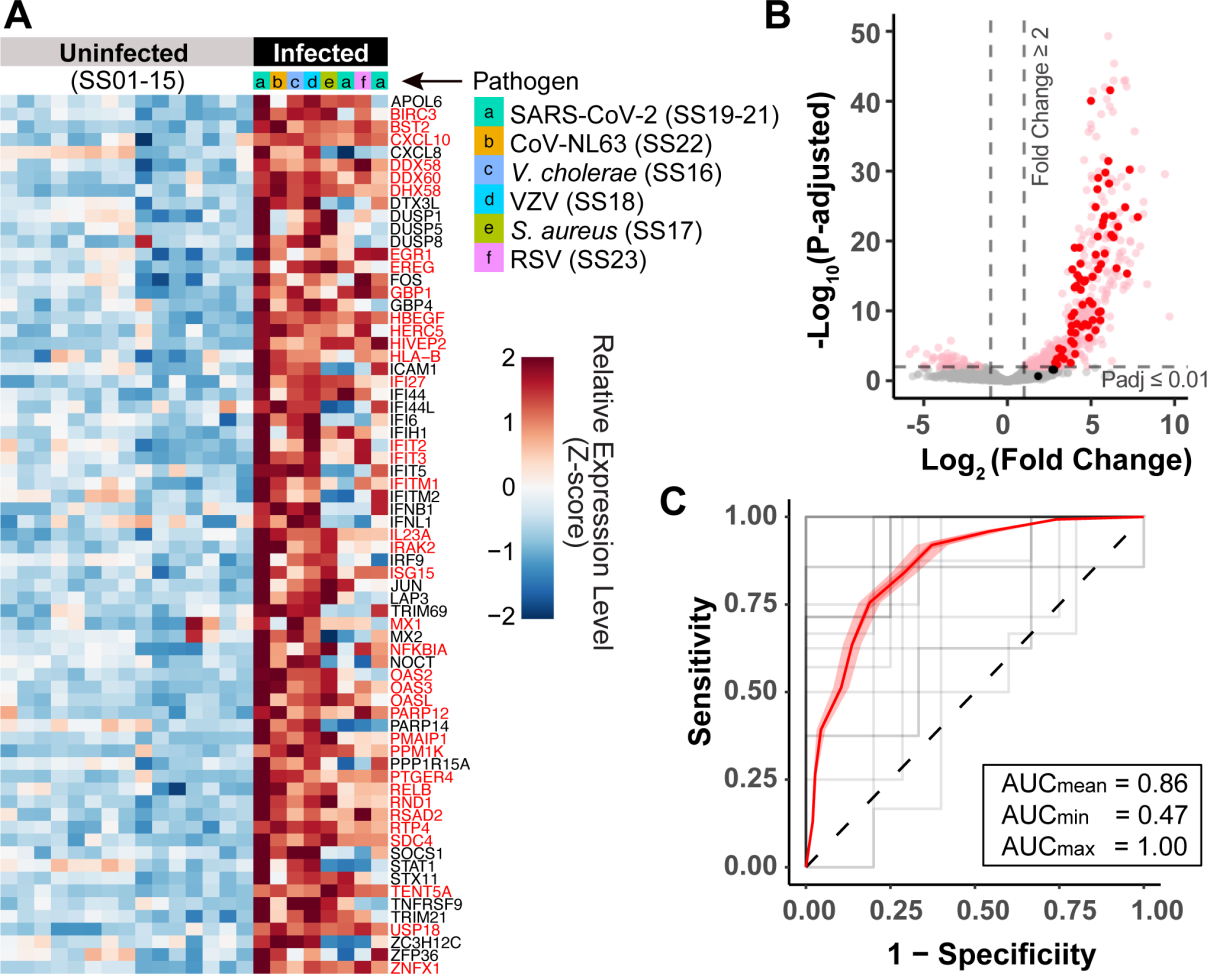
Abundance of core response transcripts in human saliva can determine whether infection is present in the body. (**A**) Heatmap showing abundance of transcripts from each of the 69 core response genes (rows) in saliva, in transcripts per million (TPM) normalized to row z-score. Each column represents the saliva sample of one individual. Genes that are significantly enriched in all eight infected individuals are highlighted in red type. (**B**) Volcano plot of all genes which were significantly upregulated in all eight infected patients compared to uninfected (DEseq2 Wald test, fold change ≥ 2, adjusted *P*-value ≤ 0.01), separated by their fold change in transcript abundance in saliva (mean of the infected vs non-infected) and Benjamini-Hochberg adjusted *P*-values. The 69 core-response genes are highlighted in dark red. (**C**) A logistic regression model was constructed with core response gene expression data from 10% of the *in vitro* data, and then used to classify whether individuals SS01–SS23 were infected just based on the core response mRNA abundance in their saliva. Gray lines indicate individual cross-validations where a random 10% of the training data were used; red line and shaded area indicate the average and variance from all 20 cross-validations, respectively.

We enumerated the reads mapping to each core response gene in the saliva of each healthy and infected enrollee (Fig. 5A). In this panel, the color scale (in z-score) indicates the number of standard deviations from the mean expression level of each gene across all individuals. As expected, the core response genes were only a subset of the 544 genes from which transcripts were significantly enriched in saliva of infected individuals (dots in Fig. 5B, adjusted *P*-value ≤ 0.01, fold change ≥ 2; Table S3). When considering all samples, transcripts mapping to 66 out of the 69 (96%) core response genes were significantly enriched in infected (all) versus healthy (all) samples (adjusted *P*-value ≤ 0.01, fold change ≥ 2). Remarkably, transcripts from 37 out of the 69 core response genes (54%) were significantly enriched in the saliva of every one of the eight infected individuals (gene names in red in Fig. 5A). We find this remarkable given so many variables between these human samples: different pathogens (including viral and bacterial), sites of infection in the body (see clinical notes in Table 2), stage of infection, and human genetic differences.

We next tested whether the abundance of core response transcripts in saliva could correctly classify if an individual is harboring an infection. A classifier trained on the expression levels of core response genes in the *in vitro* data (used in Fig. 1 to 3) correctly classified 91% of these 23 human saliva samples as having come from someone who is infected or not, based solely on the abundance of core response transcripts in their saliva. This transfer learning approach resulted in remarkably similar AUC values of 0.92 when applied to *in vitro* data in Fig. 3A versus 0.91 when applied to human saliva here. When the classifier was trained on only 10% of the *in vitro* data (a randomly selected 39 of the 387 experimental replicates from 71 studies), it could still correctly classify these 23 human saliva samples as having come from someone who is infected or healthy (Fig. 5C, mean AUC = 0.86). In conclusion, transcriptional changes observed in infected human cells in tissue culture are replicated with high fidelity in the saliva of infected humans, and a classifier built on the *in vitro* data can accurately infer whether someone is harboring an infection.

We next investigated another human cohort, in which enrollees donated saliva during the first wave of the COVID-19 pandemic on our university campus. During the 2020–21 academic year, the University of Colorado Boulder carried out weekly saliva-based SARS-CoV-2 screening for students and staff. The screening effort enabled us to enroll university affiliates into an associated human study, each of whom donated a single saliva sample used for both the university reverse transcription-quantitative PCR (RT-qPCR) test for SARS-CoV-2 (as described previously [23, 24]) and for analysis of the core response transcripts in their saliva (presented herein). For the latter analysis, we randomly chose a collection of samples from individuals who had tested positive (*n* = 48; SS33–SS80; Table 3) or negative (*n* = 20; SS81–SS100; Table 3) for SARS-CoV-2 by RT-qPCR. One feature that is unique about this cohort of 68 individuals is that all had indicated no perceptible symptoms at the time of saliva donation (23, 24).

**TABLE 3:** Saliva cohort 3, asymptomatic individuals with and without SARS-CoV-2 infection

Enrollee	Collection date	Diagnosis/infectious agent	Study site
SS33– 80	August–Dec 2020	48 SARS-CoV-2-diagnosed individuals (university affiliates) reporting no symptoms at time of saliva collection	University of Colorado Boulder
SS81– 100		20 COVID-negative and apparently healthy university affiliates	

**Fig 6:**
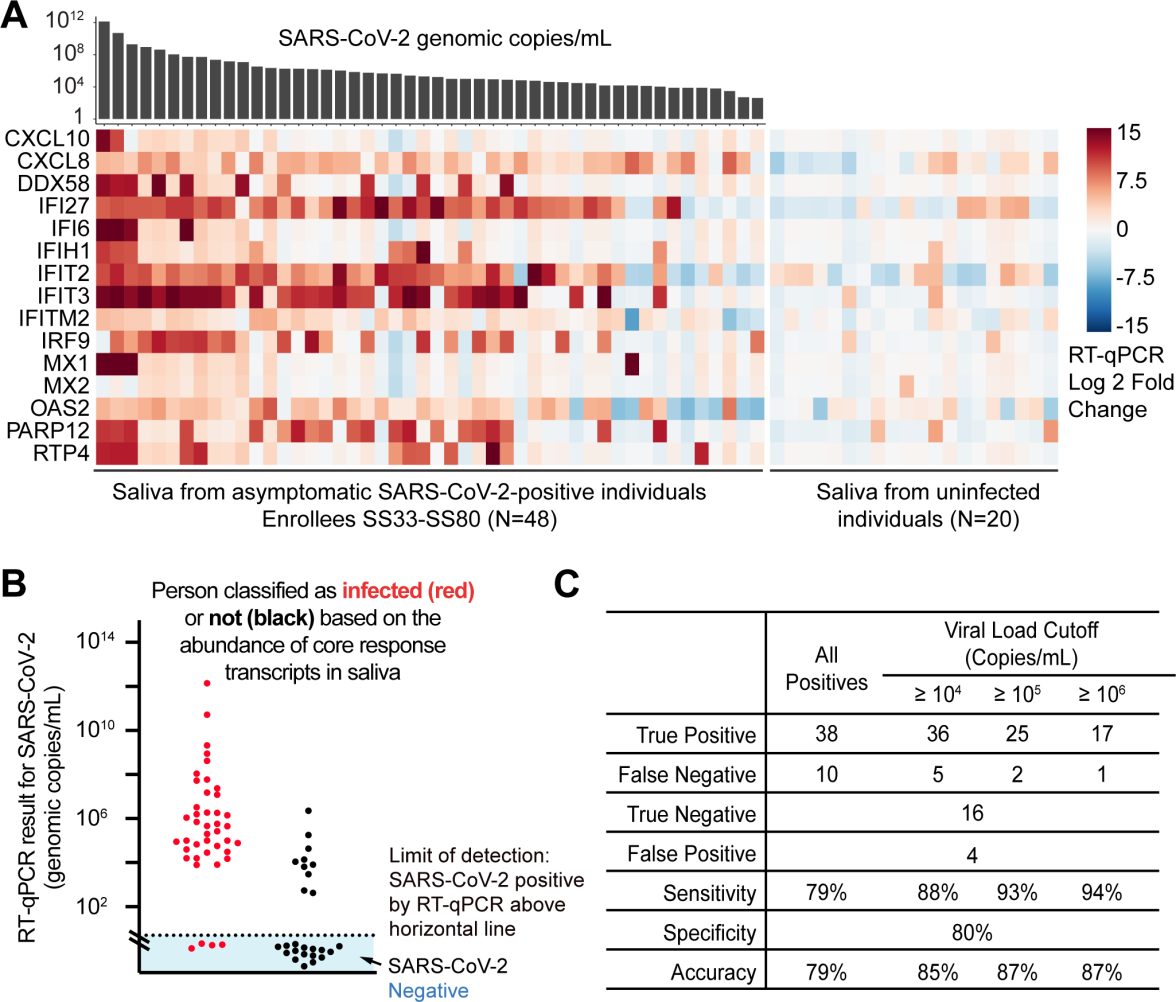
Core response transcripts are enriched in the saliva of individuals from an asymptomatic SARS-CoV-2 cohort. (**A**) Rows represent 15 human core response genes, from which transcripts were measured by RT-qPCR in a multiplex TaqMan assay. Each study enrollee is represented in a column. To derive the signal in each heatmap position, the normalized cycle threshold (Ct) value for each mRNA in that enrollee’s saliva is compared to the average normalized Ct from 20 uninfected enrollees. The SARS-CoV-2 viral load in each infected saliva sample (top of heatmap) was measured using a separate RT-qPCR assay targeting the viral genome. (**B**) We trained logistic regression models based on the core response genes’ RT-qPCR fold change data from all but one individual. We then used the model to classify the left-out individual as infected or not. This process was repeated for all individuals. The outcome of the classification using core response genes is summarized as positive (red) and negative (black), using a logistic regression probability cutoff of 0.7. Each classification was compared with the SARS-CoV-2 viral load determined using an RT-qPCR assay for the SARS-CoV-2 genome (Y-axis; as described previously [23, 24]). The SARS-CoV-2-negative individuals are represented by the dots in the blue shaded region. (**C**) A table summarizing the results in panel B, when different viral load cutoffs are considered.

We examined the transcripts from core response genes in the saliva of these individuals. For this cohort, we depart from high-throughput sequencing and use a different method to quantify core response transcripts in saliva. We developed a multiplex TaqMan RT-qPCR assay for quantifying transcripts from 15 of the core response genes, along with three control genes (Materials and Methods, Table S4). These 15 genes were chosen to represent a range of expression levels and temporal kinetics as determined using tissue culture experiments of cells infected with SARS-CoV-2 and influenza A viruses (Fig. S5 and S8). This subset of 15 genes more accurately classifies mock and infected tissue culture samples than did the original list of 69 genes, suggesting even greater classifying power by this subset of genes (Fig. S9). The abundance of these transcripts in the saliva of each enrollee is reported in Fig. 6A; Fig. S6. Overall, we observed an enrichment of transcripts from the 15 core response genes in SARS-CoV-2-positive individuals, in a manner that was positively correlated with the viral load (top of graph in Fig. 6A, highest viral loads to left).

We next trained a logistic regression model using the RT-qPCR fold change data of core response genes from all but one individual. We then classified that (left out) individual as infected or not by using the trained model and an optimal probability cutoff (Fig. S7). We did this for each individual in the cohort (Fig. 6B). Overall, we were able to identify SARS-CoV-2-positive individuals with a sensitivity of 79%, specificity of 80%, and overall accuracy of 79% (Fig. 6B and C). Importantly, we found that IFI27, IFIT2, IFIT3, CXCL8, and PARP12 are most consistently upregulated in almost all SARS-CoV-2-positive samples (Mann-Whitney U test *P*-value ≤ 0.001, comparing fold change of infected vs non-infected samples). Using only these five genes to construct logistic regression model, we are able to further improve the prediction sensitivity to 83% and accuracy to 82%. However, infectious virions are almost never recovered from individuals with viral loads below 10^6^ SARS-CoV-2 genome copies per milliliter (25–31). Individuals with viral loads below this value are either at the beginning of infection or on the long tail of recovery and are generally not thought to be infectious (32). At a cutoff of >10^6^ viral genome copies per milliliter, we were able to identify SARS-CoV-2-positive individuals with sensitivity of 94%, specificity of 80%, and overall accuracy of 87% using all 15 genes (Fig. 6C). Importantly, none of these individuals in this cohort reported symptoms at the time of saliva collection, suggesting that the host transcripts in saliva have more power to identify infection than even perceived symptoms. Thus, the abundance of core response mRNAs in saliva can correctly identify individuals in the infectious phases of SARS-CoV-2 with 87% accuracy, even though enrollees, themselves, were asymptomatic and did not know they were infected.

Finally, we interrogated the abundance of core response transcripts in saliva in the absence of infection. We enrolled seven apparently healthy individuals (cohort 4; SS26–SS32; Table 4) who were asked to collect saliva samples daily over a period of 11 days. The expression levels of the core response genes remained stable over time. When compared to day 1, transcript abundance in saliva changed no more than fivefold in subsequent days (Fig. S4). In contrast, in the SARS-CoV-2-infected patients (cohort 3, Table 3) with viral loads >10^6^ copies per milliliter, upregulation of these host mRNAs in saliva was as high as 2.0 × 10^7^ fold (mean 1.5 × 10^5^ fold) for the 15 genes measured via RT-qPCR in Fig. 6A. Thus, core response transcripts are found at steady levels in the saliva of healthy individuals but are dramatically upregulated upon infection.

**TABLE 4:** Saliva cohort 4, longitudinal saliva samples from healthy individuals

Enrollee	Collection date	Study design	Study site
SS26–32	May–Aug 2020	7 apparently healthy individuals who provided saliva samples daily for 11 days	University of Colorado Boulder

## DISCUSSION

Here, we use *in vitro* infection data to identify a small set (*n* = 69) of human “core response” genes from which transcription is upregulated by a broad variety of infections in human cell lines. We then show that these transcripts can be measured in the saliva of infected individuals (cohort 1, Table 1). The abundance of these transcripts in saliva can classify enrollees as infected or not, even when infections are caused by diverse pathogens (cohort 2, Table 2). We also show that the levels of these transcripts (from *n* = 15 of the core response genes in this case) in saliva can classify asymptomatic but infected individuals in a SARS-CoV-2 cohort (cohort 3, Table 3). In addition, finally, we show that levels of these transcripts are tightly regulated in healthy individuals who donated saliva over an 11-day time course (cohort 4; Table 4). Therefore, saliva contains molecules, in the form of host gene transcripts, which inform about the activation status of the immune system. Future efforts could, for instance, build portable detectors for these host mRNAs in saliva. Such a device could possibly replace the traditional temperature thermometer and serve as a hand-held “molecular thermometer” for infection. This could enable rapid screening and triage of large numbers of people in cases of disease outbreaks or in other contexts where infection needs to be carefully controlled.

Rather than sophisticated and complex machine learning methods or lengthy bioinformatics pipelines, this study relied only on straightforward logistic regression from mRNA abundance. Our regression-based classifications were nevertheless highly accurate, illustrating the clarity of the signal from these transcripts. For instance, rather than training our classifier on 90% of *in vitro* samples and testing it with the remaining 10%—as would be typical in 10-fold cross-validation—we instead trained using only 10% and tested on the remaining 90%. The signal from core response transcripts was also strong enough to support transfer learning in two ways: (i) we trained our classifier on samples from only two of three pathogen types (viral, bacterial, fungal) and tested on the remaining third; (ii) we trained our classifier on *in vitro* samples yet accurately classified data from human saliva biospecimens. These observations suggest a robust and consistent signal and leave open the possibility that more sophisticated classification methods could lead to even better performance in future applications.

There are two hypotheses to explain why these transcripts are found in saliva, even in the case of non-respiratory infections (Fig. 5). One possibility is that free mRNA or mRNA encapsulated in dead cells or exosomes is entering the oral cavity. This might be occurring for the purpose of targeting these structures for elimination from the body via the digestive tract. A second possibility is that interferon and other cytokines may be entering the oral cavity and stimulating cells located there to execute the transcriptional response that we are measuring (33). Currently, we cannot differentiate between the possibilities that these transcripts are transported to the mouth versus that they are produced in the mouth. Regardless, this study highlights one approach for assessing health status from saliva that extends beyond the current limited uses of saliva (for instance, in detection of oral cancers and Sjögren’s syndrome [34]). Future work should focus on parallel characterization of transcriptomics in blood and saliva so that we can grasp the full scale of systemic response to infection in different bodily fluids.

There are several limitations of this study. First, the transcriptomic studies and clinical samples included in this study represent predominantly viral infections, as viral infections are more prevalent in clinical settings and hence drew greater research focus. Although we showed that these core response genes are reliable signatures in identifying bacterial and fungal infections (Fig. 3B and 5), the sample size remains limited. Additional clinical studies are needed to further evaluate the power of these genes to classify infection across pathogen type. Second, it remains unclear whether all 69 genes together bear the most power for classifying a person as infected or not or whether a smaller subset of them would be as powerful (or even more powerful) than the full set. For instance, we found that a subset of 15 core response genes had enhanced power to distinguish infected from uninfected tissue culture samples (Fig. S9). Furthermore, 5 of these 15 core response genes showed more consistent upregulation in the SARS-CoV-2 cohort than the others (Fig. 6A). More information is also needed on the kinetics of production and decay of the core response transcripts during the time course of diverse infections in humans. This information might also refine the set of transcripts designed to optimally identify someone as infected. Also, for core response transcripts to be used for identification of infection, it will be crucial to determine the role of human genetic diversity in this transcriptional response. For instance, previous reports have shown that people with African and European ancestries elicit differential immune responses (35). However, there may be a core transcriptional response to infection shared by all or most humans, given that there is a conserved set of genes upregulated by interferon across a broad range of mammalian species as shown by the Palmarini group (36). Controlled human studies will be needed to determine how well core response genes can differentiate acute infection from other chronic medical conditions. For instance, the regulation of these core response genes in immunocompromised and individuals with auto-immune or other inflammatory diseases will need to be explored, although previous work has shown that the human genes activated by pathogens exhibit similar dynamics in immunocompromised patients as controls (37, 38). In addition, some subjects may have intra-oral inflammation due to poor or disrupted oral hygiene, a more chronic situation which may or may not result in sustained upregulation of these genes. What is presented herein is the initial concept that human acute infection might be determined from host RNA in saliva, a concept that will require additional human studies to substantiate.

## MATERIALS AND METHODS

### Analysis of *in vitro* infection data sets

We identified published data sets where human cells in culture were infected with a bacterial, viral, or fungal pathogen and where the cellular transcriptome was sequenced along with that in at least one mock-infected control. We identified a total of 71 *in vitro* infection data sets that met these criteria (Table S1). From each, we downloaded RNA-seq data sets publicly available at the NCBI SRA (short read archive) database. Raw RNA sequencing reads in FASTQ format were downloaded, trimmed using BBDuk (BBMap v38.05) (39), and mapped using HISAT2 v2.1.0 (40) to the human genome (assembly hg38). Using NCBI RefSeq genome annotation, we then counted the mapped reads assigned to genes or transcripts using FeatureCount (Subread v1.6.2) (41).

First, within each of the 71 data sets, we measured gene expression changes during infection [infected vs its matched mock control(s)]. For each individual data set, the infected replicates were compared to the corresponding mock replicates via the DESeq2 Wald test (v3.1.3) (42), from which the fold change and Benjamini-Hochberg adjusted *P*-values were obtained for each gene, correcting for multiple testing throughout. The end result was a list of genes with their relative fold change from mock in each infection study (*n* = 71), ranked by statistical significance.

Next, we identified genes that are commonly upregulated by infection across multiple studies. DESeq2 results from individual data sets were combined, and genes that are significantly upregulated (*P*_adj_ ≤ 0.05 and log_2_ fold change ≥ 1 ) in ≥80% of the data sets were extracted. As a result, we obtained 69 core response genes that satisfy the indicated criteria.

### Cross-validation using logistic regression models

To evaluate the power of the core response gene set (or gene sets from gene ontology terms) to classify infected versus uninfected conditions *in vitro* or *in vivo* from RNA-seq data sets, we built classifiers based on logistic regression models. To construct the classifiers, we extracted library size-normalized read counts in transcript per million for the 69 core response genes (or the gene ontology gene sets) from the 71 *in vitro* data sets used. Depending on the specific classifier, either all, pathogen specific, or 10% of randomly selected sequencing replicates were used to train the binomial logistic regression model using R package stats (v 3.6.2).

For cross-validation, the remaining sequencing replicates that were not used for training were subject to classification. In the case of the saliva samples from cohort 2 (Table 2), the entire data set (*n* = 23) was used for classification with the classifier trained on 10% of the *in vitro* infection data set. R package ROCR (v1.0.11) (43) was used to generate the ROC curves based on the classification outcome.

For classifying samples based on the levels of 15 of the core response genes as measured by the TaqMan RT-qPCR assay (cohort 3, Table 3), the relative fold change for each gene was calculated by first normalizing the raw Ct values to the corresponding control gene Ct (RPP30) and then comparing to the average normalized Ct of all uninfected individuals. The relative fold change values for each individual were then used for cross-validation via logistic regression. Specifically, the fold change values for all but one individual, along with their infection status, were used as the training set, while the remaining individual was subject to classification. The relationship between the classification accuracy and the probability cutoff for classifying an infected vs non-infected individual was illustrated in Fig. S7, where the optimal probability cutoff, 0.7, was used for classification in Fig. 6B and C.

### Human saliva sample collection, handling, and RNA preparation

Samples SS04, SS05, and SS12–SS21 were collected under protocol 17-0562 (University of Colorado Anschutz Medical School; PI Poeschla), where adult participants were consented verbally and donated up to 5 mL of whole saliva. Saliva was collected into Oragene saliva collection kits (DNA Genotek CP-100). The saliva is mixed with the stabilization solution in the collection kit and stored at room temperature for no longer than 2 weeks before being processed for RNA purification. Diagnosis of these individuals was provided in the form of clinical notes.

Saliva samples from individuals SS01–SS03, SS06–SS11, SS22, and SS23 were collected under protocol 19-0696 (University of Colorado Boulder, PI Sawyer), where anonymous adults verbally consented and donated up to 2 mL of whole saliva. Saliva was collected into Oragene saliva collection kit as mentioned above. For two individuals (SS22 and SS23), infection status was noticed during RNAseq procedures and ultimately determined by *in silico* metagenomic detection using GOTTCHA (v1.0b) (44) using RNAseq reads (additional RNAseq sample preparation and analysis described below). We were able to detect sequencing reads mapping to CoV-NL63 or RSV genomes from the saliva of individual SS22 and SS23, respectively, so they were presumed to be infected with these pathogens at the time of saliva collection.

Saliva samples for apparently healthy individuals over a daily time course (cohort 4, Table 4, SS26–SS32) were collected under a COVID-19-related sub-study of protocol 19-0696 (University of Colorado Boulder; PI, S. L. Sawyer), where adult participants consented verbally and donated up to 2 mL of whole saliva per day. The saliva was collected into Oragene saliva collection kit as mentioned above.

To purify RNA from saliva samples collected in Oragene saliva collection kits, we used 1 mL saliva 1:1 diluted in stabilization solution and followed the manufacturer-recommended protocol by DNA Genotek to precipitate the nucleic acid. The RNA was further DNase-digested using Turbo DNase (Invitrogen #AM2238) and cleaned up using RNA cleanup and concentration micro-elute kit (Norgen #61000). The purified RNA was used for RT-qPCR or processed further for RNA-seq.

To prepare the total RNA for sequencing, we first spiked in ERCC RNA spike-in mix (Thermo Fisher #4456740) into the saliva total RNA for downstream normalization. We depleted bacterial ribosomal RNA using pan-bacterial riboPOOL kit (siTOOLS #026). We then prepared the RNA for total RNA sequencing using KAPA RNA HyperPrep kit with RiboErase to remove human rRNA (Roche #KK8560). Finally, the saliva total RNA libraries were sequenced in 150-bp pair-end format using NovaSeq 6000 (Illumina) or in 100-bp pair-end format using BNDseq (BGI) at the depth of 30 million reads.

Saliva samples for SARS-CoV-2-infected individuals (SS33–SS80) and matched SARS-CoV-2-negative individuals (SS81-SS100) were collected under protocol 20-0417 (University of Colorado Boulder; PI, S. L. Sawyer), where adult participants 17 years of age or older (under a Waiver of Parental Consent) provided written consent. These samples were collected and tested for the SARS-CoV-2 virus during our campus COVID-19 testing initiative (23, 24) during the fall 2020, spring 2021, and summer 2021 semesters. As part of this campus testing operation, university affiliates were asked to fill out a questionnaire to confirm that they did not present any symptoms consistent with COVID-19 at the time of sample donation and to collect no less than 0.5 mL of saliva into a 5-mL screw-top collection tube. Saliva samples were heated at 95°C for 30 min on site to inactivate the viral particles for safer handling and then placed on ice or at 4°C before being transported to the testing laboratory for RT-qPCR-based SARS-CoV-2 testing performed on the same day. Samples were then kept in −80°C until RNA preparation. The total RNA of the remaining saliva samples was then purified using TRIzol LS reagent (Thermo Fisher #10296028) followed by GeneJET RNA cleanup and concentration kit (Thermo Fisher #K0841). The purified total RNA was used for RT-qPCR following the steps described below.

Additional saliva samples for general assay development were collected under protocol 20-0068 (University of Colorado Boulder; PI. S.L. Sawyer), where anonymous adult participants were verbally consented and donated up to 2 mL of whole saliva for use as a reagent in optimization and limit of detection experiments.

### Analysis of high-throughput transcriptomics data from human saliva samples

To profile human transcriptomic changes in human saliva samples, raw RNA sequencing reads in FASTQ format were obtained, trimmed using BBDuk (BBTools v38.05) (39), and mapped using HISAT2 v2.1.0 (40) to human genome assembly hg38 along with ERCC spike-in sequence reference. Using NCBI RefSeq genome annotation (GRCh38.p13), we then counted the mapped reads assigned to genes or transcripts using FeatureCount (Subread v1.6.2) (41). Read counts were first normalized using the R package RUVseq (v1.28.0) (45) to account for library size factors based on the ERCC spike-in counts. Individual samples were then separated into infected and non-infected groups, and the differential expression of genes was determined via DESeq2 (v3.1.3) Wald test (42), from which the fold change and Benjamini-Hochberg adjusted *P*-values were obtained.

### RT-qPCR analysis of core response transcripts in human saliva

Multiplexed RT-qPCR analysis for the quantitative detection of 15 of the core response transcripts was carried out using customized and multiplexed TaqMan primer and probe mixes. Together with three internal controls genes (RPP30, RACK1, and CALR), the levels of all 18 genes are measured in a total of 6 multiplexed reactions (Table S4). Understanding that the contamination of genomic DNA often introduces quantification bias when measuring host gene expression, we explicitly designed primers that span exon junctions and limit the assay elongation time so that only the host mRNA is reverse transcribed and amplified. As each transcript varies in its expression magnitude, we assigned genes into multiplex groups based on similar expression magnitudes observed in the meta-analysis of *in vitro* data sets and in human saliva. This minimizes competition of amplification reagents. Specifically, to determine the host gene expression levels, 1.5 µL of customized TaqMan multiplex probes was mixed with 5 µL 4× TaqPath 1-step multiplex master mix (Thermo Fisher # A28526), 5 µL of saliva total RNA, and 8.5 µL of nuclease-free water. The RT-qPCR assay was carried out on QuantStudio3 Real-time PCR system (Thermo Fisher) consisting of a reverse transcription stage (25°C for 2 min, 50°C for 15 min, 95°C for 2 min) followed by 40 cycles of PCR stage (95°C for 3 s, 55°C for 30 s, with a 1.6°C/s ramp-up and ramp-down rate). The cycle threshold (Ct) values were used to calculate relative fold change using delta delta Ct method. For the choice of internal control genes, we used information from the analysis of *in vitro* infected samples (Fig. 1) and the saliva RNA-seq data sets (Fig. 5; human samples) to select genes for which the expression level remained most constant and abundant across the various conditions inherent to these experiments. We optimized this TaqMan assay on RNA harvested from A549 human lung cells mock infected or infected with influenza A virus (Fig. S8).

## Data Availability

Due to issues related to donor confidentiality, raw saliva RNA sequencing data are deposited at NCBI dbGaP database, available upon request. All other original contributions presented in the study are included as supplemental material.

## SUPPLEMENTAL MATERIAL

Supplemental Material mbio.01712-23-s0001.docx Supplemental tables and figures.

ASM does not own the copyrights to Supplemental Material that may be linked to, or accessed through, an article. The authors have granted ASM a non-exclusive, world-wide license to publish the Supplemental Material files. Please contact the corresponding author directly for reuse.
