# Supplementary material for: Human mRNA in saliva can correctly identify individuals harboring acute infection
Source: mBio. 2023 Nov 9;14(6):e01712-23. doi: 10.1128/mbio.01712-23 (PMC10746177; doi:10.1128/mbio.01712-23)
Supplement: Supplemental Material — Supplemental tables and figures. [file mbio.01712-23-s0001.docx]

SUPPLEMENTAL INFORMATION

Human mRNA in saliva can correctly identify individuals harboring acute infection

Qing Yang^1, 2^, Nicholas R. Meyerson^1,3^, Camille L. Paige^1,3^, James H. Morrison^4^, Stephen K. Clark^1,3^, Will T. Fattor^1,2^, Carolyn J. Decker^5,6^, Halley R. Steiner^1,2,5^, Elena Lian^7^, Daniel B. Larremore^1,8^, Rushika Perera^7^, Eric M. Poeschla^4^, Roy Parker^1, 5, 6^, Robin D. Dowell^1,2,8, *^, Sara L. Sawyer^1, 2, *^

**Table S1.** Transcriptomics datasets used for the discovery of human core response genes

| **SRP Index** | **Human cell line** | **Pathogen** | **Abbreviation** | **Hour Post Infection** | **Sequencing Data Type** |
| --- | --- | --- | --- | --- | --- |
| SRP044763 | IMR90 | Adenovirus | ADV | 24 | mRNA |
| SRP163661 | MRC5 | Adenovirus | ADV | 24 | Total |
| SRP202003 | HepG2 | Crimean-Congo hemorrhagic fever virus | CCHFV | 72 | Total |
| SRP078309 | A549 | Dengue virus 2 | DENV2 | 36 | Total |
| SRP130978 | HUH751 | Dengue virus 2 | DENV2 | NA | Total |
| SRP132737 | Huh7 | Dengue virus 2 | DENV2 | 18 | Total |
| SRP188490 | HEK293 | Dengue virus 2 | DENV2 | 18 | Total |
| SRP101856 | DC | Ebola virus | EBOV | 24 | Total |
| SRP111145 | ARPE19 | Ebola virus | EBOV | 24 | Total |
| SRP131318 | Rhabdomyosarcoma | Enterovirus | EV | 6 | Total |
| SRP060253 | AGS | Epstein–Barr virus | EBV | NA | Total |
| SRP255890 | B Cell | Epstein–Barr virus | EBV | NA | Total |
| SRP272684 | B Cell Lymphoma | Epstein–Barr virus | EBV | 24 | Total |
| SRP212863 | HUVEC | Hantaan Orthohantavirus | HTNV | 72 | Total |
| SRP158789 | HepG2 | Hepatitis B virus | HBV | 72 | Total |
| SRP187206 | HUH751 | Hepatitis C virus | HCV | 148 | Total |
| SRP091538 | HepG2 | Hepatitis E virus | HEV | 120 | Total |
| SRP117344 | KMB17 | Herpes Simplex virus 1 | HSV-1 | 48 | Total |
| SRP154536 | HEK293 | Herpes Simplex virus 1 | HSV-1 | 4 | Total |
| SRP163661 | MRC5 | Herpes Simplex virus 1 | HSV-1 | 9 | Total |
| SRP177947 | THP1 | Herpes Simplex virus 1 | HSV-1 | 24 | Total |
| SRP189489 | HFF | Herpes Simplex virus 1 | HSV-1 | 8 | Total |
| SRP065236 | HFF | Herpes Simplex virus 2 | HSV-2 | 8 | Total |
| SRP065236 | EC | Human Cytomegalovirus | HCMV | 48 | Total |
| SRP065236 | HFF | Human Cytomegalovirus | HCMV | 48 | Total |
| SRP065236 | NPC | Human Cytomegalovirus | HCMV | 48 | Total |
| SRP163661 | MRC5 | Human Cytomegalovirus | HCMV | 48 | Total |
| SRP266618 | NTT | Human Cytomegalovirus | HCMV | 24 | Total |
| SRP065236 | CD4+ T Cell | Human Immunodeficiency virus 1 | HIV-1 | 120 | Total |
| SRP155217 | CD4+ T Cell | Human Immunodeficiency virus 1 | HIV-1 | 72 | Total |
| SRP155822 | Ileum organoid | Human Norovirus | HuNoV | 48 | Total |
| SRP223234 | HFK | Human Papillomavirus | HPV | NA | Total |
| SRP253951 | A549 | Human Parainfluenza virus 3 | HPIV3 | 24 | Total |
| SRP103819 | HNEpC | Human Rhinovirus | HRV | 48 | Total |
| SRP161185 | ATII | Influenza A virus | IAV | 24 | Total |
| SRP230823 | HeLa | Influenza A virus | IAV | 24 | Total |
| SRP234025 | A549 | Influenza A virus | IAV | 48 | Total |
| SRP253951 | A549 | Influenza A virus | IAV | 9 | Total |
| SRP272285 | A549 | Influenza A virus | IAV | 6 | Total |
| SRP277269 | 293T | Influenza A virus | IAV | 6 | Total |
| SRP281173 | A549 | Influenza A virus | IAV | 12 | Total |
| SRP170549 | Calu3 | Middle East respiratory syndrome coronavirus | MERS-CoV | 24 | Total |
| SRP227272 | Calu3 | Middle East respiratory syndrome coronavirus | MERS-CoV | 24 | mRNA |
| SRP096169 | HFF | Orf virus | ORFV | 8 | Total |
| SRP277439 | HEK293 | Porcine Rotavirus | PoRV | 12 | Total |
| SRP229586 | A549 | Respiratory Syncytial virus | RSV | 36 | Total |
| SRP229586 | H292 | Respiratory Syncytial virus | RSV | 36 | Total |
| SRP229586 | HBEC | Respiratory Syncytial virus | RSV | 36 | Total |
| SRP253951 | A549 | Respiratory Syncytial virus | RSV | 24 | Total |
| SRP115192 | HSAEpC | Rift Valley Fever virus | RVFV | 18 | Total |
| SRP094462 | HInEpC | Rotavirus | ROTAV | 6 | Total |
| SRP253951 | A549-ACE2 | Severe acute respiratory syndrome coronavirus 2 | SARS-CoV-2 | 24 | Total |
| SRP270817 | PHAE | Severe acute respiratory syndrome coronavirus 2 | SARS-CoV-2 | 48 | Total |
| SRP273473 | DC | Severe acute respiratory syndrome coronavirus 2 | SARS-CoV-2 | 2 | Total |
| SRP273473 | MAC | Severe acute respiratory syndrome coronavirus 2 | SARS-CoV-2 | 2 | Total |
| SRP278618 | iPSC-derived cardiomyocyte | Severe acute respiratory syndrome coronavirus 2 | SARS-CoV-2 | 48 | Total |
| SRP081284 | MeWo | Varicella-zoster virus | VZV | 24 | Total |
| SRP225661 | A549 | West Nile virus | WNV | 24 | Total |
| SRP142592 | hNSC | Zika virus | ZIKV | 72 | Total |
| SRP251704 | A549 | Zika virus | ZIKV | 48 | Total |
| SRP253197 | HepG2 | Zika virus | ZIKV | 48 | Total |
| SRP296743 | PBMC | *Aspergillus fumigatus* | *A. fumigatus* | 24 | Total |
| SRP296743 | PBMC | *Candida albicans* | *C. albicans* | 24 | Total |
| SRP296743 | PBMC | *Rhizopus oryzae* | *R. oryzae* | 24 | Total |
| SRP285913 | HeLa | *Chlamydia trachomatis* | *C. trachomatis* | 44 | Total |
| SRP321546 | DLD-1 | *Fusobacterium nucleatum* | *F, nucleatum* | 24 | Total |
| SRP321940 | Primary human trophoblasts | *Listeria monocytogenes* | *L. monocytogenes* | 5 | Total |
| ERP020415 | THP-1 | *Mycobacterium tuberculosis* | *M. tuberculosis* | 48 | Total |
| ERP115551 | hBMECs | *Neisseria meningitidis* | *N. meningitidis* | 6 | mRNA |
| SRP263458 | HUVEC | *Staphylococcus aureus* | *S. aureus* | 16 | Total |
| SRP072326 | A549 | *Streptococcus pneumoniae* | *S, pneumoniae* | 2 | Total |

**Table S2.** The 69 core response genes in human cell lines

| **RefSeq Accession** | **Gene Symbol** |
| --- | --- |
| NM_030641 | APOL6 |
| NM_001165 | BIRC3 |
| NM_004335 | BST2 |
| NM_001565 | CXCL10 |
| NM_000584 | CXCL8 |
| NM_014314 | DDX58 |
| NM_017631 | DDX60 |
| NM_024119 | DHX58 |
| NM_138287 | DTX3L |
| NM_004417 | DUSP1 |
| NM_004419 | DUSP5 |
| NM_004420 | DUSP8 |
| NM_001964 | EGR1 |
| NM_001432 | EREG |
| NM_005252 | FOS |
| NM_002053 | GBP1 |
| NM_052941 | GBP4 |
| NM_001945 | HBEGF |
| NM_016323 | HERC5 |
| NM_006734 | HIVEP2 |
| NM_005514 | HLA-B |
| NM_000201 | ICAM1 |
| NM_005532 | IFI27 |
| NM_006417 | IFI44 |
| NM_006820 | IFI44L |
| NM_002038 | IFI6 |
| NM_022168 | IFIH1 |
| NM_001547 | IFIT2 |
| NM_001549 | IFIT3 |
| NM_012420 | IFIT5 |
| NM_003641 | IFITM1 |
| NM_006435 | IFITM2 |
| NM_002176 | IFNB1 |
| NM_172140 | IFNL1 |
| NM_016584 | IL23A |
| NM_001570 | IRAK2 |
| NM_006084 | IRF9 |
| NM_005101 | ISG15 |
| NM_002228 | JUN |
| NM_015907 | LAP3 |
| NM_002462 | MX1 |
| NM_002463 | MX2 |
| NM_020529 | NFKBIA |
| NM_012118 | NOCT |
| NM_002535 | OAS2 |
| NM_006187 | OAS3 |
| NM_003733 | OASL |
| NM_022750 | PARP12 |
| NM_017554 | PARP14 |
| NM_021127 | PMAIP1 |
| NM_152542 | PPM1K |
| NM_014330 | PPP1R15A |
| NM_000958 | PTGER4 |
| NM_006509 | RELB |
| NM_014470 | RND1 |
| NM_080657 | RSAD2 |
| NM_022147 | RTP4 |
| NM_002999 | SDC4 |
| NM_003745 | SOCS1 |
| NM_007315 | STAT1 |
| NM_003764 | STX11 |
| NM_017633 | TENT5A |
| NM_001561 | TNFRSF9 |
| NM_003141 | TRIM21 |
| NM_080745 | TRIM69 |
| NM_017414 | USP18 |
| NM_033390 | ZC3H12C |
| NM_003407 | ZFP36 |
| NM_021035 | ZNFX1 |

**Table S3.** Top 30 differentially up- and down- regulated genes from comparison between infected and healthy saliva (cohort 3)

| **Gene Symbols** | **Log2(Fold Change)** | **Adjusted P-value** |  | **Gene Symbols** | **Log2(Fold Change)** | **Adjusted P-value** |
| --- | --- | --- | --- | --- | --- | --- |
| **CHRNA5** | 6.05 | 9.35E-76 |  | **LOC102723665** | -3.38 | 1.86E-06 |
| **IL2RA** | 6.07 | 1.08E-71 |  | **GCSAM** | -4.12 | 1.84E-05 |
| **STS** | 6.02 | 7.91E-69 |  | **TAAR9** | -5.50 | 2.94E-05 |
| **BAG5** | 5.80 | 9.31E-64 |  | **CDCA7L** | -3.59 | 1.16E-04 |
| **HBD** | 7.01 | 3.53E-53 |  | **MIR320B2** | -4.81 | 1.47E-04 |
| **POR** | 6.03 | 4.83E-50 |  | **HULC** | -5.84 | 1.49E-04 |
| **LCN10** | 6.38 | 4.06E-46 |  | **ZNF235** | -3.25 | 2.40E-04 |
| **C10orf55** | 7.06 | 9.76E-44 |  | **SLC39A12** | -3.05 | 3.28E-04 |
| **TWIST1** | 6.35 | 1.08E-43 |  | **IVNS1ABP** | -3.87 | 3.58E-04 |
| **CA2** | 6.97 | 1.19E-43 |  | **KLHDC4** | -3.96 | 4.01E-04 |
| **NR0B1** | 7.13 | 7.96E-43 |  | **SERPINB5** | -3.57 | 4.41E-04 |
| **GALE** | 5.83 | 1.04E-42 |  | **LOC101927143** | -4.42 | 4.45E-04 |
| **TENT5A** | 6.15 | 2.69E-42 |  | **VAV2** | -3.29 | 4.68E-04 |
| **WRN** | 5.11 | 3.91E-42 |  | **DSEL** | -4.39 | 5.69E-04 |
| **NOS3** | 5.95 | 5.09E-41 |  | **RPL22** | -2.67 | 7.18E-04 |
| **HBEGF** | 5.00 | 8.94E-41 |  | **LINC01085** | -3.48 | 7.23E-04 |
| **DRD4** | 6.13 | 5.62E-40 |  | **ERVW-1** | -3.94 | 8.02E-04 |
| **NCMAP** | 6.31 | 3.29E-39 |  | **SLC25A25-AS1** | -3.54 | 8.58E-04 |
| **REN** | 5.61 | 7.10E-39 |  | **THOC5** | -2.59 | 9.56E-04 |
| **FGG** | 4.98 | 2.07E-37 |  | **UXT-AS1** | -4.49 | 1.21E-03 |
| **HADHA** | 5.01 | 8.57E-37 |  | **TRI-AAT1-1** | -3.34 | 1.37E-03 |
| **HBG2** | 7.61 | 2.11E-36 |  | **AKAP4** | -3.07 | 1.76E-03 |
| **HOXD13** | 4.86 | 2.50E-36 |  | **TADA2A** | -2.58 | 2.03E-03 |
| **KITLG** | 5.31 | 1.18E-35 |  | **LRRC7** | -3.49 | 2.71E-03 |
| **CHRNB1** | 5.74 | 1.08E-32 |  | **LEMD1-AS1** | -3.55 | 3.02E-03 |
| **ITGB3** | 4.59 | 2.63E-32 |  | **GNG14** | -3.82 | 3.37E-03 |
| **BST2** | 6.03 | 3.66E-32 |  | **ZNF461** | -3.55 | 3.77E-03 |
| **OR56B1** | 7.34 | 4.66E-31 |  | **LINC01781** | -2.66 | 4.07E-03 |
| **HBG1** | 8.01 | 5.45E-31 |  | **SAMD13** | -3.46 | 4.65E-03 |
| **RND1** | 7.31 | 6.27E-31 |  | **SLAMF8** | -1.81 | 5.00E-03 |

**Table S4.** Multiplex TaqMan RT-qPCR assay for monitoring host immune gene signature expression.

| **Group** | **Gene Target** | **Primer Name** | **Primer sequence (5’->3’)** | **Probe Sequence (5’->3’)** | **Probe Dye** |
| --- | --- | --- | --- | --- | --- |
| **1**  **(Controls)** | CALR | CALR_F | GAGTATTCTCCCGATCCCAGTATCTATGCC | ATGAGGCATACGCTGAGGAGTTTGG | ABY |
|  |  | CALR_R | ATTTGTTTCTCTGCTGCCTTTGTTACGCCC |  |  |
|  | RACK1 | RACK1_F | TCCCACTTTGTTAGTGATGTGGTTATCTCC | CAGTTTGCCCTCTCAGGCTCCT | VIC |
|  |  | RACK1_R | CAAATCGCCTCGTGGTGGTGCCCGTTGTGAG |  |  |
|  | RPP30 | RPP30_F | AGATTTGGACCTGCGAGCG | TTCTGACCTGAAGGCTCTGCGCG | FAM |
|  |  | RPP30_R | GAGCGGCTGTCTCCACAAGT |  |  |
| **2** | DDX58 | DDX58_F | CCGGAAGACCCTGGACCCTA | TTAGGGAGGAAGAGGTGCAG | ABY |
|  |  | DDX58_R | AGGGCATCCAAAAAGCCACG |  |  |
|  | IFIT2 | IFIT2_F | CCCTGCCGAACAGCTGAGAA | CTGCAACCATGAGTGAGAAC | VIC |
|  |  | IFIT2_R | AGTTGCCGTAGGCTGCTCTC |  |  |
|  | IFITM2 | IFITM2_F | ATAGCATTCGCGTACTCCGT | TGCCTCCACCGCCAAGTGC | FAM |
|  |  | IFITM2_R | TGATGCCTCCTGATCTATCGC |  |  |
| **3** | Mx1 | Mx1_F | TAGAGAGCTGCCAGGCTTTG | TACACACCGTGACGGATATG | ABY |
|  |  | Mx1_R | ATCTGTGAAAGCAAGCCGGA |  |  |
|  | IFI6 | IFI6_F | TCGCTGCTGTGCCCATCTATC | CTGCTGCTCTTCACTTGC | VIC |
|  |  | IFI6_R | TTCTTACCTGCCTCCACCCCAC |  |  |
|  | IFIT3 | IFIT3_F | ACAGCAGAGACACAGAGGGCA | TCATGAGTGAGGTCACCAAG | FAM |
|  |  | IFIT3_R | AGCTGTGGAAGGATTTTCTCCAGG |  |  |
| **4** | IFI27 | IFI27_F | GCCACGGAATTAACCCGAGC | CATCAGCAGTGACCAGTGTG | ABY |
|  |  | IFI27_R | GCCACAACTCCTCCAATCACA |  |  |
|  | IFIH1 | IFIH1_F | ACAGCTTCACCTGGTGTTGGA | CGAAGCAAGCCAAAGCTGAAG | VIC |
|  |  | IFIH1_R | ATGGCAAACTTCTTGCATGGCT |  |  |
|  | PARP12 | PARP12_F | ACCATGCAAACCTGCAATACC | TCCAGGCCCGAAGAGCATC | FAM |
|  |  | PARP12_R | GCAGCGTGCGGTTAAAGAG |  |  |
| **5** | IRF9 | IRF9_F | GCTCTTCAGAACCGCCTACTTC | CTCCAGCCATACTCCACAGAATC | ABY |
|  |  | IRF9_R | CTCCAGCAAGTATCGGGCAA |  |  |
|  | CXCL10 | CXCL10_F | TGCAAGCCAATTTTGTCCACG | AGCAGTTAGCAAGGAAAGGTC | VIC |
|  |  | CXCL10_R | GCCTCTGTGTGGTCCATCCT |  |  |
|  | Mx2 | Mx2_F | CATGATTGTGAAGTGCCGGG | CTGAGCTTGGCAGAGGCAAC | FAM |
|  |  | Mx2_R | CAACGGGAGCGATTTTTGGA |  |  |
| **6** | OAS2 | OAS2_F | CGTTGGTGTTGGCATCTTCTG | CCAGTCCCATCCTTGAAGCAG | ABY |
|  |  | OAS2_R | TGCATTGTCGGCACTTTCC |  |  |
|  | CXCL8 | CXCL8_F | CCAGGAAGAAACCACCGGAA | TGGCCGTGGCTCTCTTG | VIC |
|  |  | CXCL8_R | CTTGGCAAAACTGCACCTTCAC |  |  |
|  | RTP4 | RTP4_F | TGGACGCTGAAGTTGGATGGC | CTCTCTGTTGGTATTGCTTC | FAM |
|  |  | RTP4_R | CAACTTCGCTGGCAGGAGGAA |  |  |


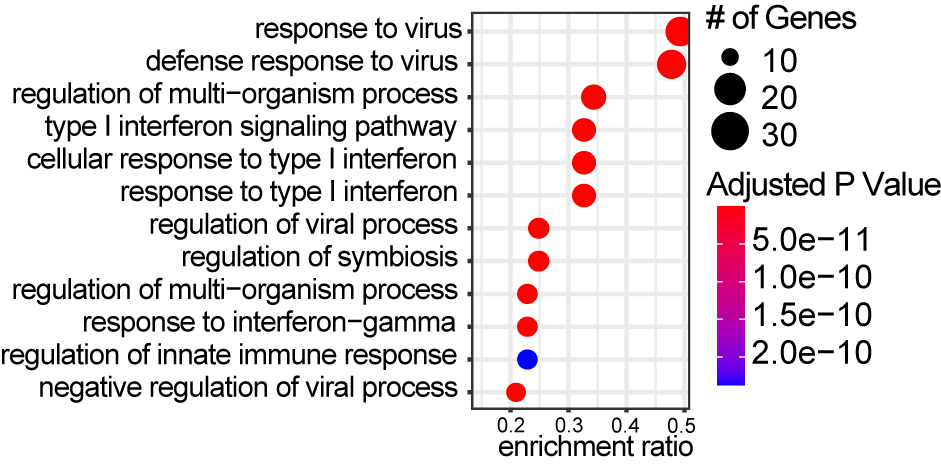


**Figure S1. Characterization of the identified core response genes via gene ontology enrichment analysis.** The X-axis, enrichment ratio, is the number of observed genes divided by the number of expected genes in each gene ontology (GO) category. The adjusted P-value indicates the probability of observing the given number of genes in each category by chance. Functions related specifically to antiviral responses are the most enriched, possibly due to an over representation of viruses within the datasets analyzed in panel A, or because innate immunity to viruses is better studied and therefore the genes involved are better annotated.


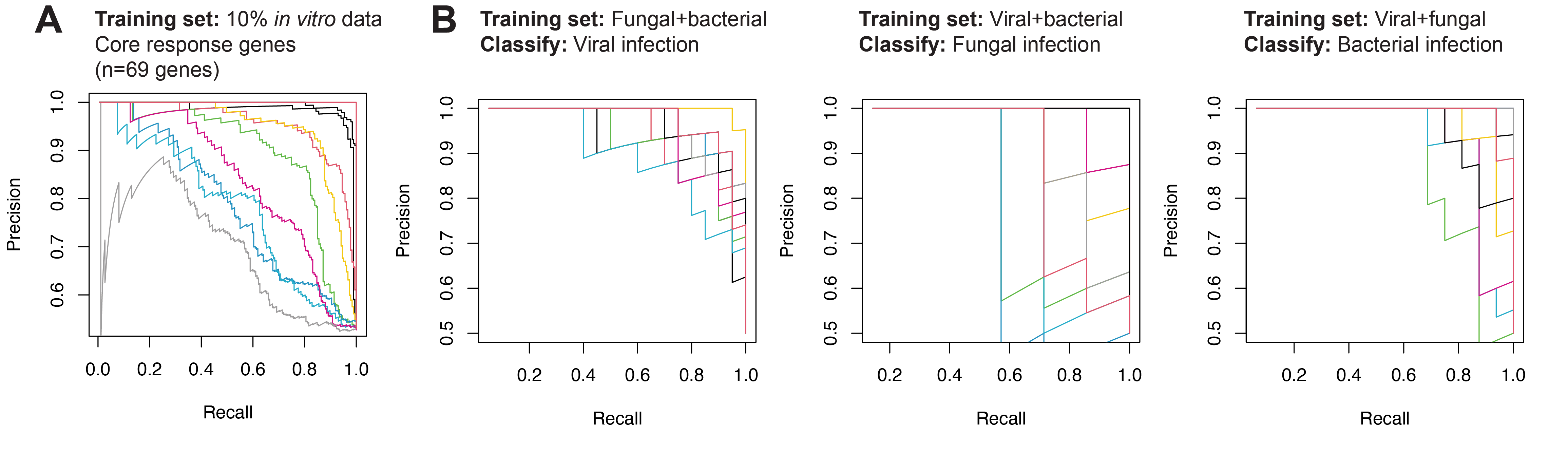


**Figure S2. Precision-recall curves of the various infection classifiers.** Various logistic regression classifiers were established using the expression levels of the 69 core response genes. The precision against the recall for each classifier performance is summarized in each graph. Each cross validation is repeated 10 times, indicated by different colors. (A) A logistic regression model was trained on data from 10% of the 387 samples from the 71 in vitro datasets, then used to classify the other 90% of the samples as mock-infected or infected. (B) Cross validation analyses between different types of infections. In each case, the logistic regression model was trained on infections of two types (top of graph) and used to classify human cells as having been infected or not with the third type of pathogen, based solely on the expression level of the core response genes.


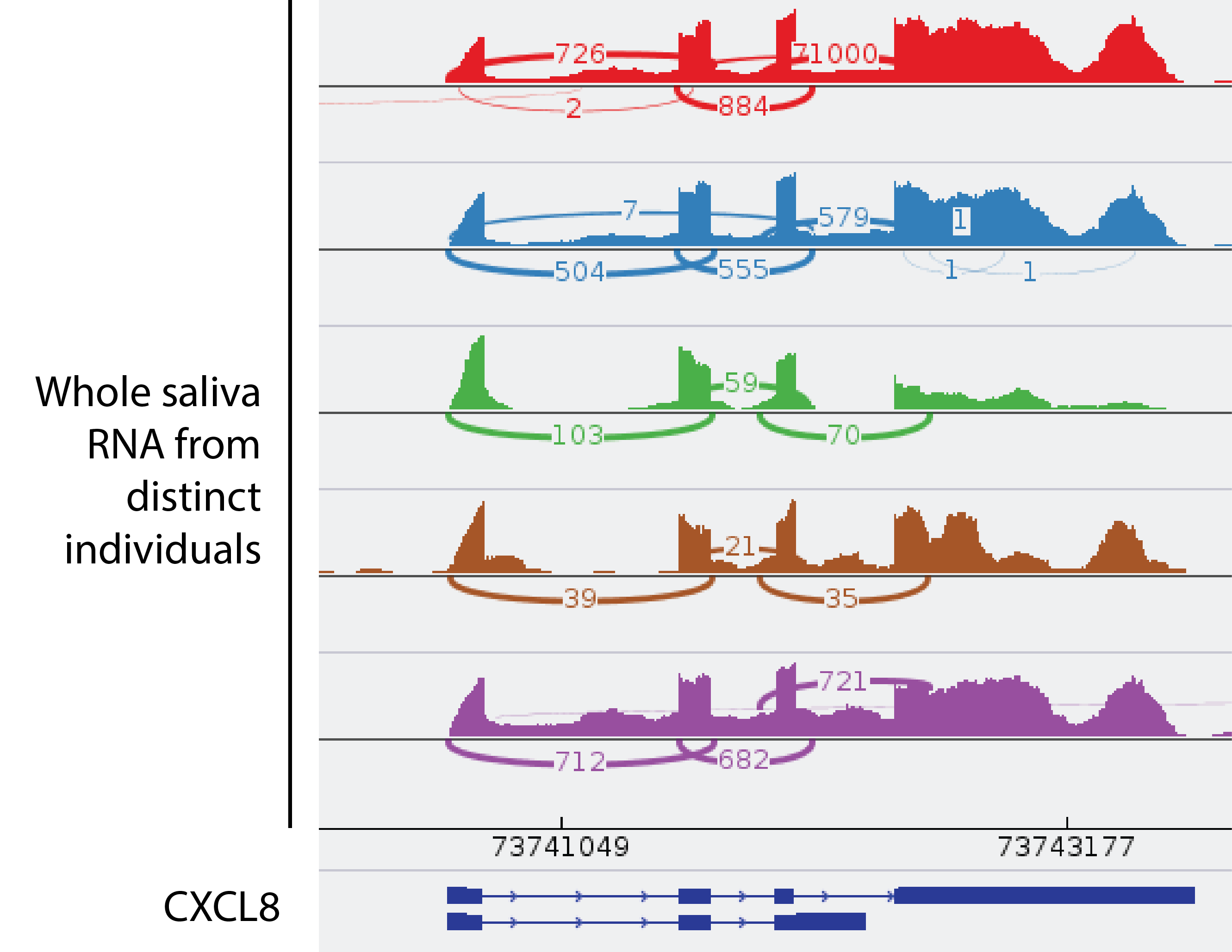


**Figure S3. mRNA structure is preserved in human saliva samples.** Sashimi plot indicating mRNA structure is preserved during the saliva sample processing and collection, so that the exon regions are preferentially sequenced over the introns. Shown here are saliva samples from 5 individuals, CXCL8 gene is selected as the example.


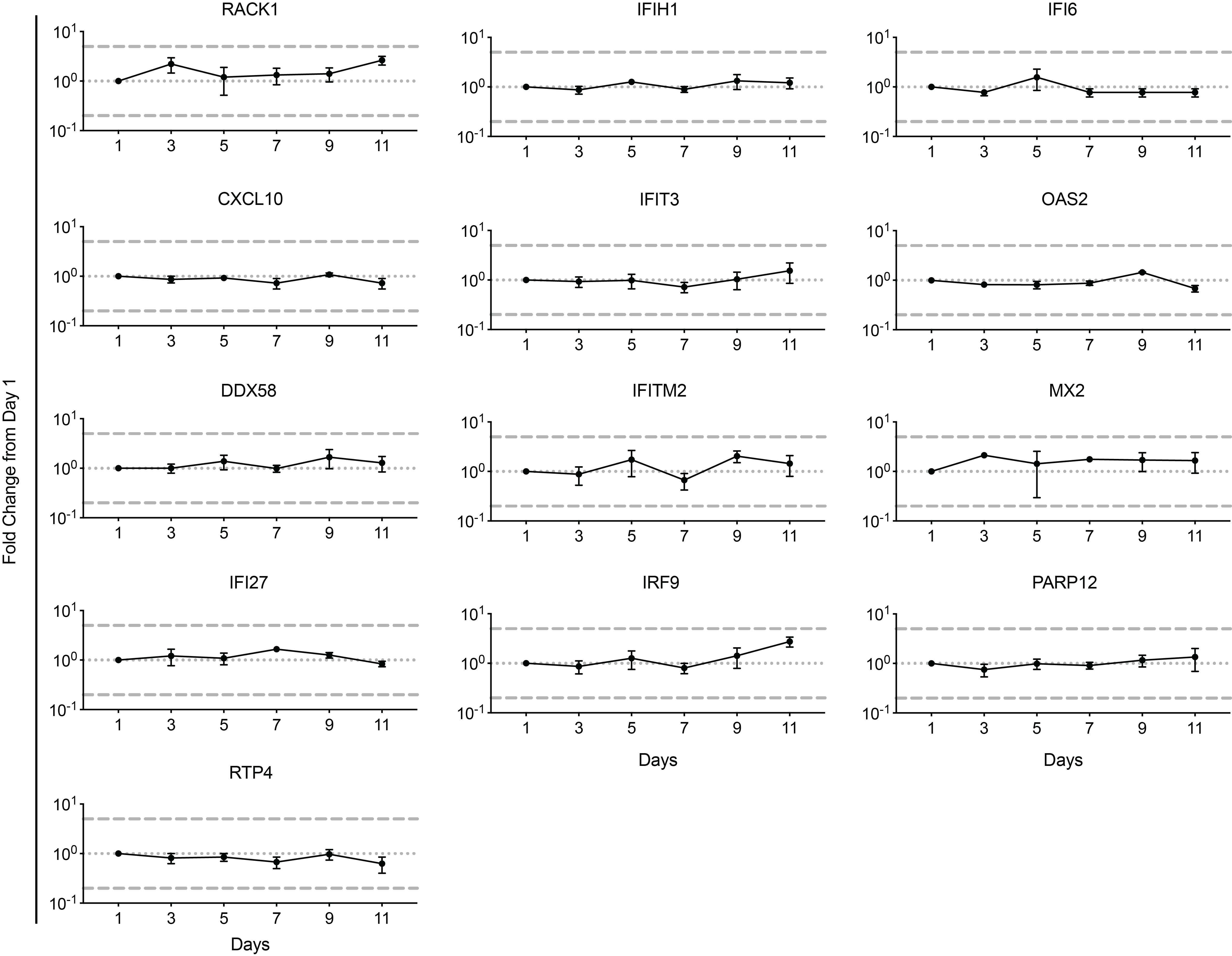


**Figure S4. Relative fold change of core response genes over time in healthy human saliva.** To determine the extent of mRNA variation from day to day in human saliva samples, 7 individuals (SS26-SS32) were asked to collect saliva on daily basis over a period of 11 days. Total RNA was isolated from each sample and used as a template in the multiplex TaqMan assay also used on the asymptomatic SARS-CoV-2 cohort described in the manuscript. Shown here are data from 1 control gene (RACK1) and 12 core response genes (IFIH1, IFI6, CXCL10, IFIT3, OAS2, DDX58, IFITM2, MX2, IFI27, IRF9, PARP12 and RTP4). Error bars represent the SEM of 7 individuals. In all panels, Ct value is converted to fold change by normalizing the Ct value to the Ct value of RPP30, and then normalized again to the abundance of mRNA measured on Day 1 for each individual.


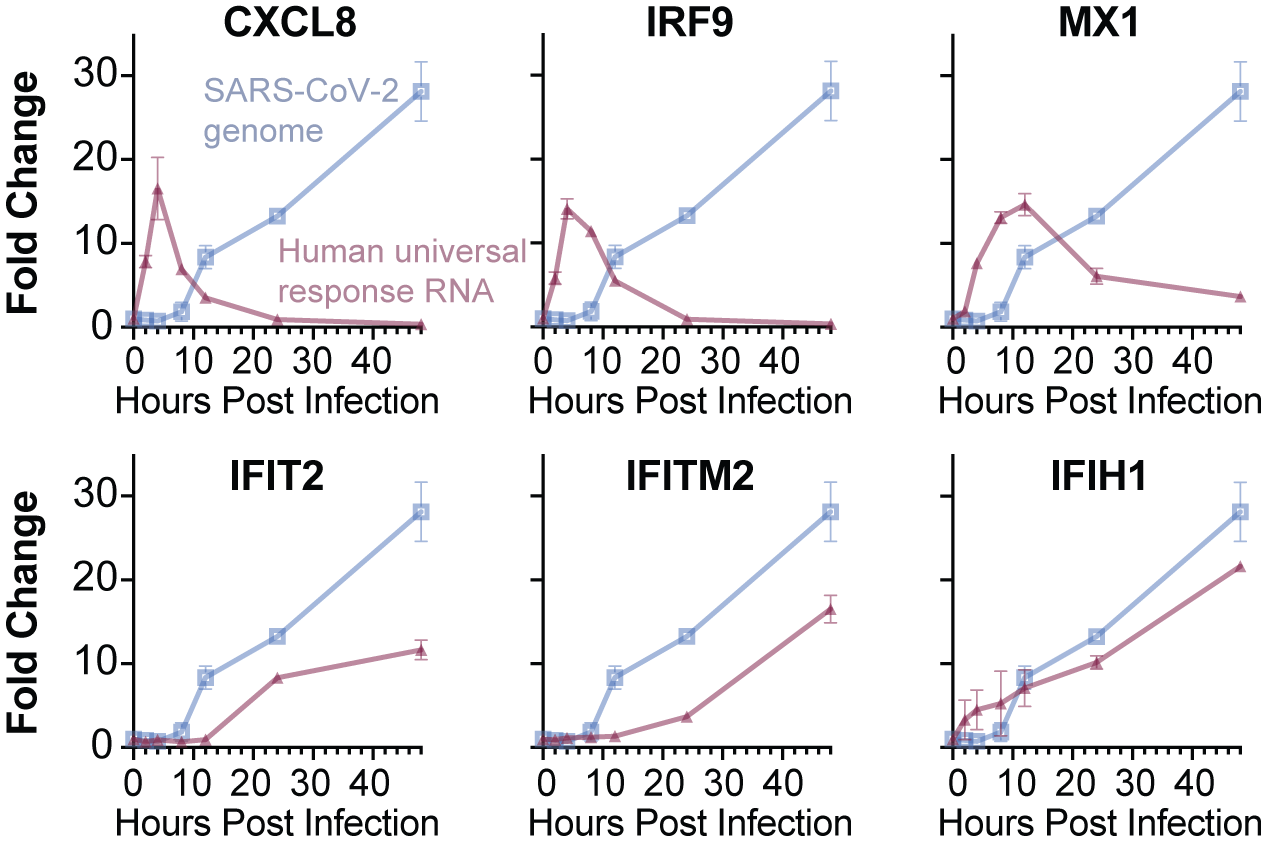


**Figure S5. Core response genes are up- and down-regulated with different kinetics upon infection.** Huh7 human liver cells were infected with SARS-CoV-2 at MOI of 0.01 over a time course of 48 hours. Total RNA was harvested 0, 2, 4, 8, 12, 24, and 48 hours post infection. The fold changes of six core response transcripts (top of each graph; red data line) and of the SARS-CoV-2 genome (blue data line) were measured by multiplexed TaqMan RT-qPCR assay (see Method). Error bars represent the SEM of 3 biological replicates. Ct value is converted to fold change by normalizing the Ct value to the Ct value of RPP30, and then normalized again to the abundance of mRNA measured in a mock infection. Some core response genes (CXCL8, IRF9, MX1) are upregulated in the early time points of the infection and then rapidly downregulated within the first 24 hours. This is quite interesting, since this is a low-MOI spreading infection and new cells are constantly getting infected. This would be consistent with a pulse of activity that is then quickly downregulated by a feedback loop. On the other hand, the upregulation of other core response genes (such as the classical type-I interferon inducible genes, IFIT2, IFITM2, and IFIH1), starts later and increases steadily along with viral genome replication. This result suggests that the abundance of mRNA from any specific core response gene will depend on the timepoint during infection, even in situations of spreading infections as would be the case in the human body.

Methods: Human Hepatoma (Huh7) cells (gift from Charles Rice, Rockefeller University) were grown in 1X DMEM (ThermoFisher cat. no. 12500062) supplemented with 2 mM L-glutamine (Hyclone cat. no. H30034.01), non-essential amino acids (Hyclone cat. no. SH30238.01), and 10% heat inactivated Fetal Bovine Serum (FBS) (Atlas Biologicals cat. no. EF-0500-A). The virus strain used for the assay was SARS-CoV2, USA WA 01/2020, passage 3. Virus stocks were obtained from BEI Resources and amplified in Vero E6 cells to Passage 3 (P3) with a titer of 5.5 × 10^5^ PFU/mL. Cells were resuspended to 6.0 × 10^5^ cells/mL in 10% DMEM and seeded at 2 mL/well in 6-well plates. The plates were then incubated for approximately 24 hours (h) at 37°C, 5% CO_2_ for cells to adhere prior to infection. Cells were infected with SARS-CoV-2 at an MOI of 0.01. Samples were harvested at 0, 2, 4, 8, 12, 24, and 48 hours post infection in 200 μl TRIzol reagent for RNA extractions following the manufacture’s protocol.

**
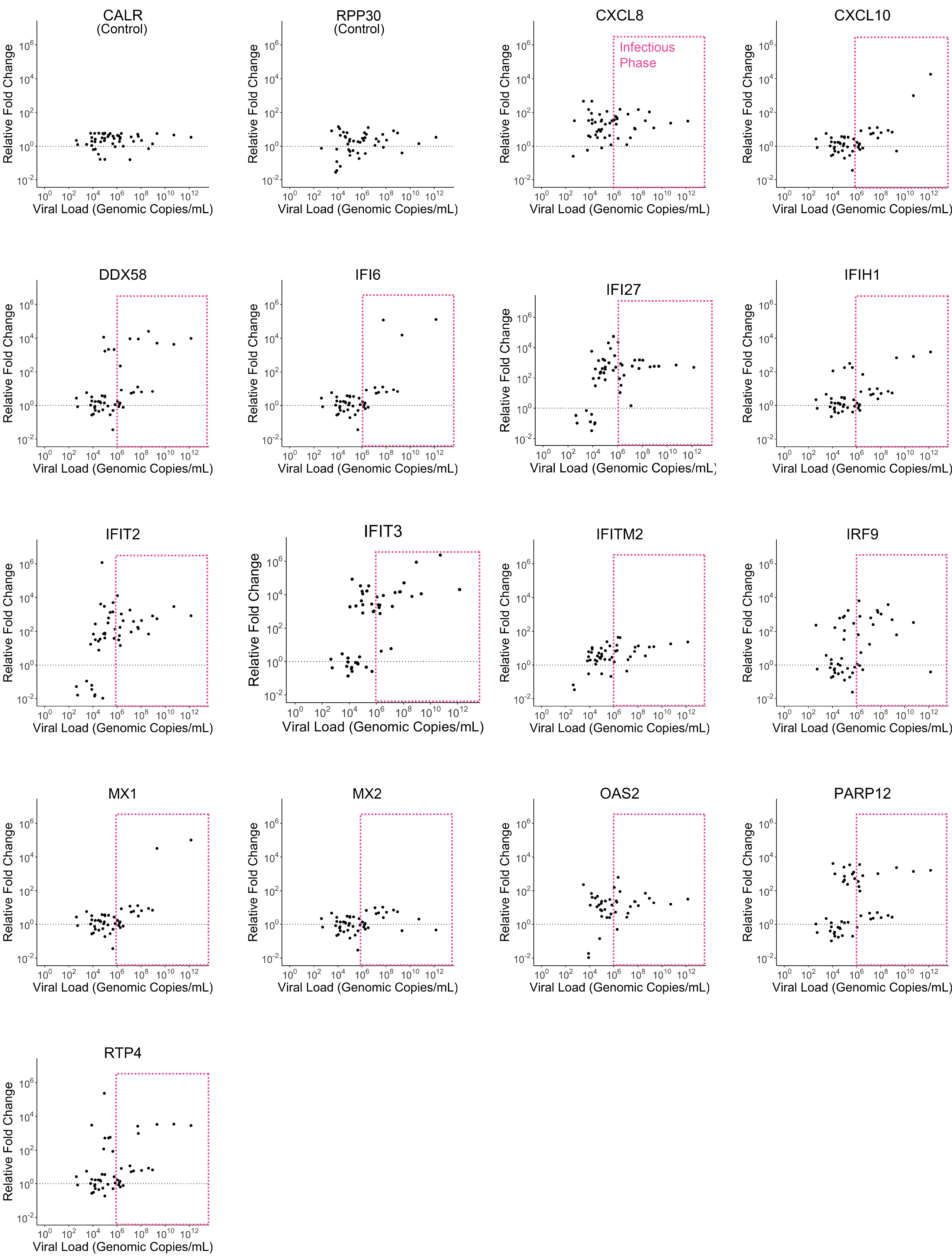
**

**Figure S6. Abundance of core response mRNA in human saliva correlates with relative viral load in saliva samples from SARS-CoV-2+ individuals.** For core response genes, we plotted the relative fold change of core response mRNA in saliva (Y axis) against the concentration of viral genome copies in saliva (X axis). The X axis corresponds to SARS-CoV-2 viral load, determined by RT-qPCR**.** The Y axis shows the relative fold change of the human mRNA noted at the top of the graph, determined by the TaqMan RT-qPCR assay described in the methods. Each measurement of human mRNA was compared to the average of the same measurement from the saliva of 20 uninfected samples, to calculate the relative fold change that is shown. The horizontal dashed line indicates the fold change of 1. A pink box shows the range of viral loads where people are considered infectious (above 10^6^ viral copies/mL (1–7)). This is because infectious virions are almost never recovered from individuals with viral loads below 10^6^ viral copies per mL (1, 2, 4–8). Individuals with lower viral loads are either at the beginning of infection, or on the long tail of recovery (9). Interestingly, the core response transcripts accumulate in saliva before this point, at the transition of viral titers to above 10^4^ viral copies/mL. This is consistent with a model where these transcripts accu­­mulate in saliva specifically during, and possibly before, periods of acute viral replication.


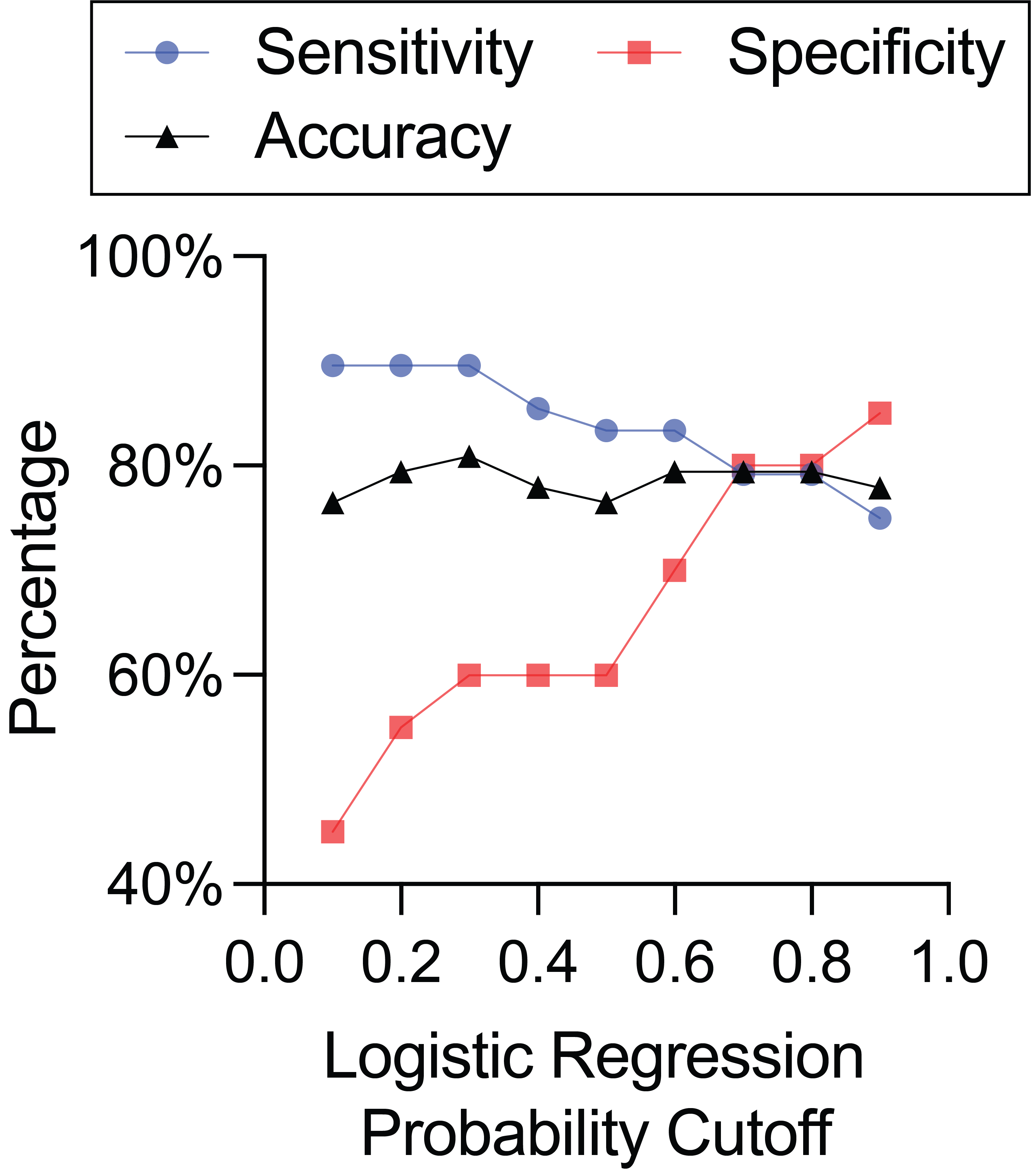


**Figure S7. Relationship between the core response screening performance and the probability cutoff for the leave-one-out logistic regression model.** In order to assess the performance of the infection screening using the core response genes, we trained logistic regression models based on the RT-qPCR fold change data from all but one individual from the asymptomatic SARS-CoV-2 cohort (SS33-SS100). We then used the model to classify whether the one individual was infected or not, given a probability cutoff from 0.1 to 0.9 (x-axis). This process is repeated among all individuals, and the classifications made were then compared with the SARS-CoV-2 infection condition determined using the pathogen-specific RT-qPCR assay. The relationship between the probability cutoffs and the comparison outcomes, including specificity (red), sensitivity (blue), and accuracy (black), are summarized in the figure above.


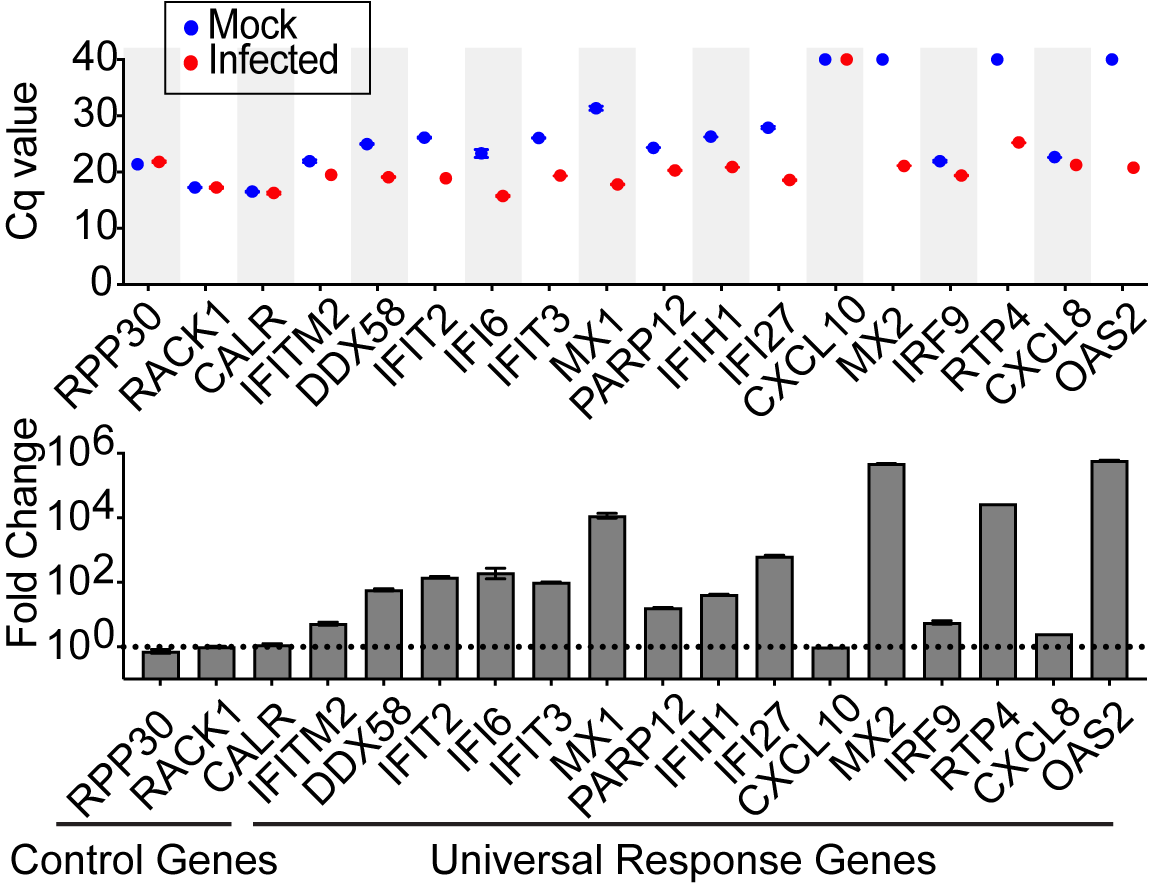


**Figure S8. Optimization of TaqMan assay in cells infected with influenza A virus.** A549 human lung cells were infected with Influenza A virus at multiplicity of infection (MOI) of 0.1 for 24 hours. Total RNA was harvested from the cells and 100 ng was used as template in the multiplex TaqMan assay described. To demonstrate the dynamic range and the signal consistency, the raw Ct values are shown in the top panel, and the resulting fold changes are shown in the bottom panel. The error bar indicates the SEM from 2 biological replicates. Ct value is converted to fold change by normalizing the Ct value to the Ct value of RPP30, and then normalized again to the abundance of mRNA measured in a mock infection.

Methods: We optimized this TaqMan assay on RNA harvested from A549 human lung cells mock infected or infected with influenza A virus (H3N2/Udorn/307/72) at MOI of 0.1 for 24 hours. Human lung epithelial cells (A549s) where plated at a concentration of 1x10^6^ cells/well in a 6-well plate. The next day, the cells were infected with influenza A virus at an MOI=0.1 in serum-free media containing 1.0% bovine serum albumin. After 1 hour incubation, the inoculum was removed and replaced with growth media containing 1 ug/mL of N-acetylated trypsin. 24 hours post-infection, total RNA was harvested using QIAGEN RNeasy Mini kit (QIAGEN #74104). Using these samples, we confirmed that the assay can measure each mRNA over a large dynamic range (Ct 15-40) with small amount of input RNA (≥100 ng). At this moderate MOI and relatively short infection timepoint, already 14 out of the 15 measured genes are upregulated. The range of mRNA upregulation in infected cells ranged from 2.6-fold (CXCL8) to 6.1x10^5^-fold (OAS2).


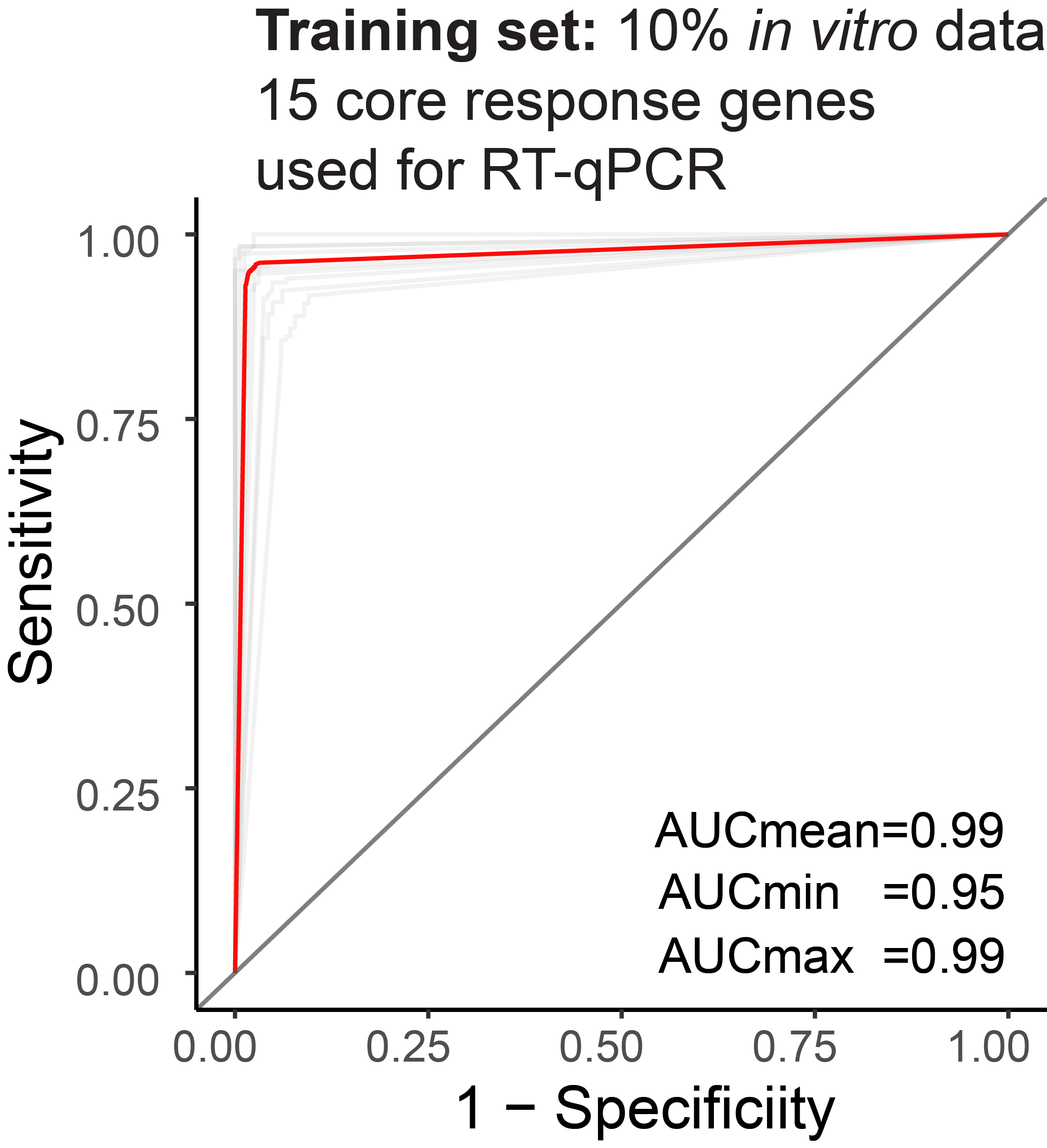


**Figure S9. Performance of an infection classifier based on the 15 core response genes used in the RT-qPCR panel.** Receiver operating characteristic (ROC) curves of the logistic regression model established using the expression levels of the 15 selected core response genes used for RT-qPCR (Figure 6). The area under curve (AUC) is summarized in each graph. Each cross validation is repeated 10 times, with grey lines indicating each replicate of cross validation; red curve summarizes the average ROC curve. A logistic regression model was trained on data from 10% of the 387 samples from the 71 *in vitro* datasets, then used to classify the other 90% of the samples as mock-infected or infected.

**References**

1. Arons MM, Hatfield KM, Reddy SC, Kimball A, James A, Jacobs JR, Taylor J, Spicer K, Bardossy AC, Oakley LP, Tanwar S, Dyal JW, Harney J, Chisty Z, Bell JM, Methner M, Paul P, Carlson CM, McLaughlin HP, Thornburg N, Tong S, Tamin A, Tao Y, Uehara A, Harcourt J, Clark S, Brostrom-Smith C, Page LC, Kay M, Lewis J, Montgomery P, Stone ND, Clark TA, Honein MA, Duchin JS, Jernigan JA. 2020. Presymptomatic SARS-CoV-2 Infections and Transmission in a Skilled Nursing Facility. New Engl J Med <https://doi.org/10.1056/nejmoa2008457>.

2. Huang C-G, Lee K-M, Hsiao M-J, Yang S-L, Huang P-N, Gong Y-N, Hsieh T-H, Huang P-W, Lin Y-J, Liu Y-C, Tsao K-C, Shih S-R. 2020. Culture-Based Virus Isolation To Evaluate Potential Infectivity of Clinical Specimens Tested for COVID-19. J Clin Microbiol 58.

3. Wölfel R, Corman VM, Guggemos W, Seilmaier M, Zange S, Müller MA, Niemeyer D, Jones TC, Vollmar P, Rothe C, Hoelscher M, Bleicker T, Brünink S, Schneider J, Ehmann R, Zwirglmaier K, Drosten C, Wendtner C. 2020. Virological assessment of hospitalized patients with COVID-2019. Nature 1–10.

4. Scola BL, Bideau ML, Andreani J, Hoang VT, Grimaldier C, Colson P, Gautret P, Raoult D. 2020. Viral RNA load as determined by cell culture as a management tool for discharge of SARS-CoV-2 patients from infectious disease wards. Eur J Clin Microbiol 39:1059–1061.

5. Jaafar R, Aherfi S, Wurtz N, Grimaldier C, Hoang VT, Colson P, Raoult D, Scola BL. 2020. Correlation Between 3790 Quantitative Polymerase Chain Reaction–Positives Samples and Positive Cell Cultures, Including 1941 Severe Acute Respiratory Syndrome Coronavirus 2 Isolates. Clinical Infectious Disease.

6. Bullard J, Dust K, Funk D, Strong JE, Alexander D, Garnett L, Boodman C, Bello A, Hedley A, Schiffman Z, Doan K, Bastien N, Li Y, Caeseele PGV, Poliquin G. 2020. Predicting infectious SARS-CoV-2 from diagnostic samples. Clin Infect Dis 71:ciaa638-.

7. Kim M-C, Cui C, Shin K-R, Bae J-Y, Kweon O-J, Lee M-K, Choi S-H, Jung S-Y, Park M-S, Chung J-W. 2021. Duration of Culturable SARS-CoV-2 in Hospitalized Patients with Covid-19. New Engl J Med 384:669–671.

8. Wölfel R, Corman VM, Guggemos W, Seilmaier M, Zange S, Müller MA, Niemeyer D, Jones TC, Vollmar P, Rothe C, Hoelscher M, Bleicker T, Brünink S, Schneider J, Ehmann R, Zwirglmaier K, Drosten C, Wendtner C. 2020. Virological assessment of hospitalized patients with COVID-2019. Nature 581:465–469.

9. Savela ES, Winnett A, Romano AE, Porter MK, Shelby N, Akana R, Ji J, Cooper MM, Schlenker NW, Reyes JA, Carter AM, Barlow JT, Tognazzini C, Feaster M, Goh Y-Y, Ismagilov RF. 2021. Quantitative SARS-CoV-2 viral-load curves in paired saliva and nasal swabs inform appropriate respiratory sampling site and analytical test sensitivity required for earliest viral detection. Medrxiv 2021.04.02.21254771.
